# Causal parametric language mapping with electrical stimulation during awake neurosurgery

**DOI:** 10.1126/sciadv.adw1599

**Published:** 2026-02-25

**Authors:** J. Raouf Belkhir, Frank E. Garcea, Eduardo Navarrete, Benjamin L. Chernoff, Max H. Sims, Sam Haber, Arka N. Mallela, Matthew Pease, Susan O. Smith, Eric B. Hintz, Thandar Aung, Eliza M. Reedy, Kevin Walter, Tyler Schmidt, Jorge Gonzalez-Martinez, Nduka Amankulor, Webster H. Pilcher, Bradford Z. Mahon

**Affiliations:** ^1^ Department of Psychology, Carnegie Mellon University, Pittsburgh, PA, USA.; ^2^ School of Medicine, University of Pittsburgh, Pittsburgh, PA, USA.; ^3^ Neuroscience Institute, Carnegie Mellon University, Pittsburgh, PA, USA.; ^4^ Department of Neurosurgery, University of Rochester Medical Center, Rochester, NY, USA.; ^5^ Dipartimento di Psicologia dello Sviluppo e della Socializzazione, Università di Padova, Padova, Italy.; ^6^ MindTrace Technologies Inc., Pittsburgh, PA, USA.; ^7^ Department of Neurosurgery, Albany Medical Center, Albany, NY, USA.; ^8^ Department of Neurosurgery, University of Pittsburgh Medical Center, Pittsburgh, PA, USA.; ^9^ Department of Neurosurgery, Indiana University, Indianapolis, IN, USA.; ^10^ Department of Neurosurgery, Penn Medicine Lancaster General Health, Lancaster, PA, USA.; ^11^ Department of Neurosurgery, University of Pennsylvania, Philadelphia, PA, USA.

## Abstract

Functional mapping with direct electrical stimulation (DES) is widely used during awake neurosurgery to generate causal evidence about person-specific neuroanatomical organization. According to a long-standing clinical and scientific paradigm, if the application of DES to a given brain region does not result in performance errors, that site is considered to be uninvolved in the task. Here, we show that both error rates and performance speed on correct trials are parametrically modulated by when DES starts and stops relative to the timeline of task-driven processing in stimulated brain areas and networks. We propose a framework, causal parametric mapping, which moves beyond the classic approach of binarizing the effects of DES on behavior into “positive” and “negative” mapping trials. Causal parametric mapping offers a method to functionally dissect separable processing stages in the human brain, in real time, with reversible causal evidence during invasive neurosurgical procedures.

## Teaser

Temporal parameters of direct electrical stimulation reveal dissociable stages of language processing.

## INTRODUCTION

Functional mapping with direct electrical stimulation (DES) is an invasive neurosurgical technique used to define person-specific neurobiological organization of sensory, motor, and cognitive functions. During DES, small amperages of electrical current (1 to 15 mA) are applied for brief periods of time (seconds) at discrete sites in the brain while a patient is performing a neurocognitive task (*1*). If performance is disrupted by electrical stimulation, the stimulated region is considered necessary for task performance, and the surgery is tailored to avoid that region, as is possible given other clinical constraints. Because DES mapping provides person-specific, causal evidence about functional brain organization in real time, it has been the clinical standard for over half a century to guide real-time surgical resection decisions during brain tumor and epilepsy surgery (*1*–*9*).

Picture naming, or visual naming, has long been the standard task used with DES to map language (*2*, *4*). Repeatable picture-naming errors, caused by DES at a given site in the brain, constitute positive evidence that the stimulated site is involved in language (*10*, *11*). Errors in picture naming caused by DES include hesitations, dysfluencies, failures to respond, and, more rarely, overt paraphasic errors such as phonoarticulatory, phonological, or semantic errors (*7*, *11*, *12*). Prior research indicates that the absence of errors across multiple trials with DES can be considered positive evidence the stimulated region is “safe” to remove (*7*). Generally, the rate of errors caused by DES is between 5 and 10% (*7*, *13*, *14*)—meaning that over 90% of picture-naming trials with DES lead to a correct response with no apparent delay. For instance, a landmark study (*7*) found that 69% of tested patients (129/186) did not have positive language sites for picture naming (defined by DES-induced errors) within the confines of tailored craniotomies over the temporal lobe. Given the language functions commonly ascribed to the temporal lobe, this raises the question of whether the exclusive reliance on DES-induced errors could underestimate the role of some brain regions in language processing. Studies using extracranial (magnetic or direct current) stimulation have demonstrated functional dissociations using response times —both in the domain of language and in other domains of cognition (*15*–*17*).

Ballistic language tasks such as picture naming involve a rapid progression of processing across functionally and anatomically separable stages. We thus reasoned that the timing parameters of when DES starts and stops, relative to the underlying processing stages in the stimulated sites, may modulate how DES affects behavior. Here, we show that (i) physical parameters of DES modulate both the likelihood that DES will cause an error and performance speed on correct trials and (ii) patterns of DES-induced modulations of performance speed on correct trials map onto perisylvian regions known to support separable processing stages in word retrieval.

### Reevaluating how direct electrical stimulation can be used to map function

Since the inception of DES as a clinical tool, clinical and scientific orthodoxy has been that only DES-induced errors represent “true-positive” language sites. Assuming that baseline error rates on trials without DES (false alarms) are low, this means that sensitivity of DES to map language function is based (exclusively) on errors. Trials for which DES is applied and the patient is correct, with no noticeable hesitation in response time, are considered “true negatives.” If DES to a site does not cause errors, that site is inferred to not be involved in the task the patient is performing.

The endurance of DES as a clinical tool is a testament to the simplicity and directness of the method for supporting real-time decisions about the localization of function in a specific person’s brain. Prior discussions have highlighted many parameters that collectively modulate how DES affects behavior, including the amplitude and frequency of stimulation, the location of application, and the task being performed by the participant relative to the region being mapped (*4*, *5*, *18*, *19*). However, even holding those parameters constant, DES to the same region, in the same patient, performing the same task, can lead to variable effects across trials. In some cases, DES to a given site in a patient will invariably cause an error. However, it is also often the case that DES to a given site will sometimes, but not always, cause errors during a mapping session (*8*, *20*). Why there is such variability is not understood. That uncertainty has led some to question the value of the method for delineating the neurobiological organization of language (*8*). Here, we show that by integrating performance metrics across both correct and incorrect trials, all of the data generated during awake mapping can be used toward the goal of functional mapping.

Our approach, although novel in its application to DES, draws on established approaches for integrating speed and accuracy to constrain inferences about dissociable processing stages (*21*–*23*). Dual-task logic (*24*–*26*) provides a framework for testing how temporal parameters of DES affect functionally separable stages of processing. Dual-task paradigms are based on the premise that if a neurocognitive process is transiently blocked, delayed, or otherwise occupied, then downstream processes will be delayed as a function of when the block started as well as the duration of the block (*24*, *25*). We propose that electrical stimulation can act to transiently block processing in the stimulated region and its connected network, causing a delay for downstream processes and ultimately for the (correct) spoken utterance. The magnitude of that disruption is determined, at least in part, by the physical parameters of the DES train, including its timing, duration, amplitude, and location. Transient disruptions to behavior caused by DES could be modeled as the addition of noise to a specific processing stage, prolonging the time required for that stage to resolve its computation or converge on the target representation.

Word retrieval cued by pictures involves a two-stage process (*27*, *28*) that is organized across dissociable perisylvian language regions. Posterior lateral temporal and posterior inferior parietal regions support the relatively early stage(s) of lexical semantic processing [for review, see (*29*)]. By contrast, the anterior supramarginal gyrus, pars opercularis, and pre– and post–central sensorimotor regions support later stages of phonological encoding and phonoarticulatory and laryngeal planning and production (*30*). Although different models debate the degree to which lexical-semantic and phonological-articulatory processing stages overlap in time (*27*, *28*, *31*), all models of visual naming agree that lexical semantic processing architecturally precedes phonoarticulatory processing.

### The current project

**Fig. 1.**
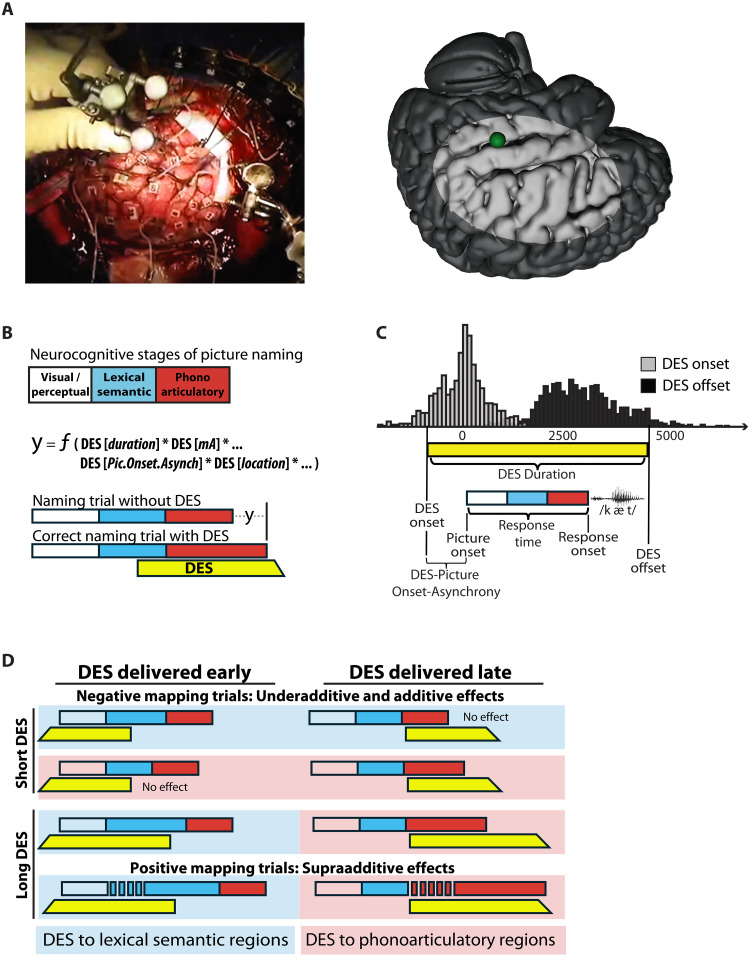
Causal parametric mapping of processing stages in language production. (**A**) Example DES trial visualized from overhead camera in the operating room (left) and on template brain (green sphere; right). (**B**) Schematic of neurocognitive stages during picture naming (visual, lexical semantic, and phonoarticulatory) and how physical parameters of DES are modeled to relate to behavioral effects. (**C**) Histograms show the distributions of DES onset and offset times across all DES events in all patient participants. The schematic illustrates variables computed for each DES event relative to picture and response onset for each naming trial. (**D**) Conceptual framework illustrating how DES timing (early versus late delivery), duration, and location modulate naming performance across processing stages. Underadditive effects are observed on (some) negative mapping trials (no clinically significant deviation in performance), whereas supraadditive effects correspond to positive mapping trials (hesitations and speech errors). Blue and red shading denotes DES applied to regions supporting lexical semantic and phonoarticulatory stages of processing, respectively.

Because separable processing stages in language production are anatomically and temporally dissociated, we leveraged the natural variability generated by the clinical procedure of awake mapping to test whether physical parameters of DES modulate its effectiveness to disrupt behavior. We first show that physical parameters of DES affect both the likelihood of making an error and performance speed on correct trials, and that there is anatomical alignment between the effects on error likelihood and on performance speed. We then reasoned that if, in the course of producing a word, DES transiently blocks a process supported by a stimulated region, the resulting delay in speech production will be proportionate to (i) when the disruption began and (ii) how long it lasted (*32*). Accordingly, two core predictions are tested:

First, during a visual naming task, the disruption of performance caused by DES will be modulated by when DES is applied relative to picture onset (see Fig. 1). If DES is applied too late in time to a region supporting an early stage of processing (lexical semantic access), DES will have little opportunity to affect behavior. This is because processing will have largely “passed” the stage of processing supported by that region when DES was applied. Similarly, if DES is applied too early to a region supporting a late stage (e.g., phonoarticulatory processing), it is predicted to have a reduced effect on behavior because the effectiveness of DES will have dissipated by the time the region’s computations are engaged by the task. Thus, DES is predicted to maximally affect behavior when it is applied at the right time relative to when the stimulated brain region is engaged in task processing (Fig. 1, A and B).

The second core prediction is that the covariance between the duration of DES and response onset times on correct trials will index the degree to which the stimulated region constitutes a functional bottleneck on processing. For instance, phonoarticulatory processes represent a single channel bottleneck: Only one speech sound can be produced at a time, and at the anatomical level, late-stage phonoarticulatory processes are supported by circumscribed perisylvian regions. This generates the expectation that, when DES is applied to regions supporting phonoarticulatory processes, a 100-ms increase in the duration of DES will lead to a 100-ms increase in the (correct) response time to the picture. By contrast, the duration of DES is predicted to have an underadditive effect on response onset time when it affects processes that are implemented in parallel over anatomically distributed regions—such as lexical semantic processing (*27*, *29*, *33*, *34*).

## RESULTS

### Parametric modulation of speed and accuracy by DES

Analyses were based on 2498 picture-naming trials during awake brain surgery across 19 patients (tables S1 and S2; Materials and Methods). A total of 262 trials were excluded from analysis due to interruptions unrelated to stimulation (e.g., patient talking with clinicians). Of the 2236 trials included in analysis, 39% (*n* = 867) were paired with DES to the cortical surface; trials that were not paired with DES (*n* = 1369) served as a within-session baseline across analyses to control for general effects on speed of the intraoperative context (semilateral positioning, recent sedation, medications, and existing pathology). The variables on which our analyses were focused were all parameters that were under control of the clinical team and were modulated for clinical purposes: DES amplitude (mA), location in the brain, timing relative to picture onset, and duration of stimulation. On average, DES was applied 6 ms before picture onset (SD = 651 ms) and had a duration of 2903 ms (SD = 1031 ms; Fig. 1A). Response time analyses excluded error trials, as well as correct trials with response times that were statistical outliers (see Materials and Methods), ensuring that all response time effects reflect the central tendency of correct performance. On a subset of correct trials (7%; 52/745), patients initiated their responses only after the DES train had ended. Although included in accuracy analyses (i.e., considered “correct”), that 7% of trials was excluded from the primary response time analyses and analyzed separately.

**Fig. 2.**
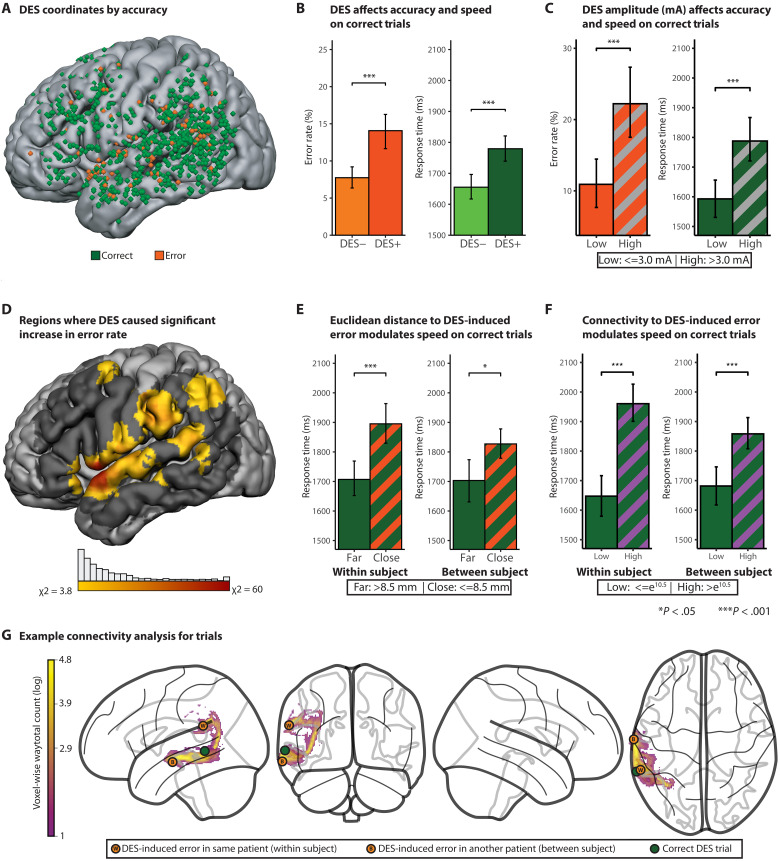
DES parameters have parallel effects on accuracy and response times on correct trials. (**A**) Anatomical locations (coordinates) of DES across all patients were reconstructed via real-time cranial navigation, confirmed with intraoperative video review, and then transformed into MNI space (MNI ICBM 152) for group-level analyses. (**B**) Across all stimulated regions, DES increased the likelihood of naming errors (χ2 = 22.5; *P* < 0.001) and slowed response times on correct or negative mapping trials [Welch *t* test: *t*(1770) = 4.22, *P* < 0.0001] relative to nonstimulated trials. (**C**) Trials with higher DES amperage were associated with an increased likelihood of errors (chi-square analysis: χ2 = 12.6; *P* < 0.0004) and longer response times on correct trials [*t*(378) = 3.93, *P* < 0.0002] relative to nonstimulated trials. (**D**) A searchlight analysis showed that DES increased the likelihood of errors (relative to baseline) in perisylvian structures, including the inferior frontal gyrus (IFG), vSMC, temporal pole, supramarginal gyrus, angular gyrus, superior temporal gyrus (STG), and middle frontal gyrus (MFG) (χ2 > 3.8; *P* < 0.05). (**E**) Response times on correct trials were slower when DES was administered closer (measured with Euclidean distance) to a DES-induced error site, both within-subject [*t*(553) = 4.04, *P* < 0.0001] and between-subject [*t*(488) = 2.85, *P* < 0.005]. (**F**) Probabilistic tractography was computed between each DES coordinate from correct trials, and the nearest error in the same patient (within-subject analysis) as well as the nearest error in a different patient (between-subject analysis). Higher connectivity between correct trials and their respective maximally connected error sites was associated with slower response times in both the within-subject and between-subject analyses. (**G**) Example probabilistic tractography from a DES coordinate in the middle temporal gyrus that did not cause an error (green) to a DES coordinate that did cause an error, within-subject (orange W), and between-subject (orange B).

Across all trials with DES, there was an 82% increase in the likelihood of an error compared to nonstimulated trials in the same naming session: 14.1% with DES (122/867) versus 7.7% without DES (106/1369) (Fig. 2B; χ^2^ = 22.5; *P* < 0.0001). Because patients undergoing awake brain surgery can have elevated baseline error rates (in the absence of stimulation), we estimated the DES-specific error rate by subtracting the error rate on nonstimulated trials (7.7% of 867 = 67 errors) from the rate on trials with DES (122 errors), yielding an adjusted DES-induced error estimate of ~6.9% [(122 − 67)/(867 − 67)]. This value aligns well with prior reports (*7*, *14*). Figure 2A displays the stimulation locations for each DES event, distinguishing DES-induced errors (orange coordinates) from correct trials paired with DES (green coordinates). As a first indication that DES can affect performance on correct trials, we found that response times on correct trials were, overall, slower on trials paired with DES compared to trials without DES [*t*(1770) = 4.22, *P* < 0.0001].

To identify regional effects of DES on behavior, we developed a “searchlight analysis” that aggregates local error rates and response times across a cube of 125 voxels (5 by 5 by 5 voxels, each voxel is 3-mm isotropic, for a total volume per searchlight of 3375 mm^3^). Searchlight cubes, centered at each voxel of the brain, were constrained to regions with at least 10 DES events across the sample of patients (fig. S1). This analysis revealed significantly elevated error rates in perisylvian language regions compared to baseline (see Fig. 2E for details; threshold set at χ2 > 3.8; *P* < 0.05).

We then tested whether DES amperage (mA) modulated both accuracy and performance speed on correct trials. For the accuracy analysis, higher DES amplitude was associated with a higher error rate (Fig. 2C; χ2 = 12.6; *P* < 0.0004). This observation is in line with prior observations (*5*, *10*, *17*) and underlines the importance of setting DES amplitudes appropriately. The same pattern was present for response times on correct trials: A median split based on stimulation amplitude on correct trials with DES (median = 3.5 mA; Fig. 2C) showed that higher stimulation amplitudes were associated with longer response times [*t*(378) = 3.93, *P* < 0.0002]. Higher stimulation amplitudes cause more current spread, which may increase the likelihood for disruption of both local and nonlocal processes (e.g., higher stimulation amplitudes may propagate further through functional networks) (*13*, *35*).

### Testing for an intrinsic relation between performance speed and DES-induced errors

A general expectation of causal parametric mapping is that there will be anatomic alignment between patterns of accuracy and patterns of performance speed on correct trials. One way to test this is to evaluate whether the proximity of DES coordinates on correct trials to locations of DES-induced errors modulates performance speed on those correct trials. “Proximity” in the brain can be measured in two broad ways. One measure of proximity is physical distance, for instance, as measured with the Euclidean distance between two coordinates in the brain. A second approach is to measure the connectivity between two coordinates. Prior research (*8*, *36*, *37*) indicates that electrical stimulation spreads both via general volume conduction (physical distance measured with Euclidean distance) and by propagating down white matter pathways [“connectivity distance,” measured with probabilistic tractography, computed over diffusion magnetic resonance imaging (MRI) data]. We therefore tested whether increased proximity (physically closer, higher connectivity) between DES coordinates from correct trials and coordinates of DES-induced errors is associated with slower correct response times.

We computed both the Euclidean and connectivity-based distances between each DES coordinate from each correct trial and the nearest coordinate associated with a DES-induced error. This was carried out separately for within-subject and between-subject analyses. For within-subject analyses, the distances (physical, connectivity) between each correct trial with DES to the nearest error in that subject were calculated. For between-subject analyses, we computed the distances (physical, connectivity) between each correct trial and the nearest error (in any other subject). Between subject analyses allowed us to ask whether proximity (physical and connectivity) generalizes across patients. Subjects who did not make errors (*n* = 4) were excluded from within-subject analyses but retained in between-subject analyses.

#### Euclidean distance

For the within-subject analysis, a median split analysis over Euclidean distances (median = 8.5 mm) revealed that response times were significantly slower on trials for which the DES site was relatively close to a DES-induced error (<8.5 mm), in the same patient, compared to trials that were comparatively far (>8.5 mm; Fig. 2D; *t* = 4.04, *P* < 0.0001). This effect remained significant after controlling for DES amplitude, in both linear and rank-based regression models (*P* < 0.015 and *P* < 0.004, respectively). The same pattern emerged in the between-subject analysis: Response times were significantly slower on trials that were relatively close to the nearest DES-induced error (<8.5 mm) compared to trials that were relatively far from the nearest DES-induced error (>8.5 mm; *t* = 2.85, *P* < 0.005), in any other subject. These data demonstrate anatomic coupling between errors and response times on correct trials, indicating that performance speed on “negative mapping” trials is sensitive to the same underlying functional processes that are identified by DES-induced errors.

#### Connectivity

Parallel analyses were conducted to test whether connectivity between DES coordinates from correct trials to those for DES-induced errors is related to performance speed on correct trials. We computed subject-specific structural connectivity between each correct DES coordinate and the most strongly connected DES-induced error site using probabilistic tractography. Each correct trial was thus assigned the connectivity value reflecting the strongest structural link to an error-associated region (fig. S2). In both within-subject and between-subject analyses, higher maximum connectivity predicted slower response times [within-subject linear: *R*^2^ = 0.039, *F*(1,517) = 21.2, *P* < 0.0001; rank-based: ρ = 0.27, *P* < 0.0001; between-subject linear: *R*^2^ = 0.032, *F*(1,641) = 21.1, *P* < 0.0001; rank-based: ρ = 0.15, *P* < 0.0001].

#### Integrated analyses of Euclidean distance and connectivity

As expected, variance in Euclidean distance was inversely related to variance in connectivity (*R*^2^ = 0.393, *P* < 0.0001): Regions that are close tend to have higher connectivity, as measured with probabilistic tractography. We tested whether each distance measure accounted for separable variance. Nested model comparisons showed that, for the within-subject analysis, connectivity explained variance over and above Euclidean distance (nested model: *F* = 15.43, *P* < 0.0001), whereas Euclidean distance did not explain additional variance beyond connectivity (*F* = 1.11, *P* = 0.29). In contrast, in the between-subject analyses, both Euclidean distance and connectivity had independent contributions to performance speed (connectivity: *F* = 26.99, *P* < 0.0001; distance: *F* = 8.44, *P* < 0.004). These findings suggest that structural connectivity is the primary measure of “distance” relevant for understanding how the location at which DES is applied will affect behavior. More broadly, and in line with a core premise of causal parametric mapping, these findings indicate an intrinsic anatomic relation between performance speed on correct trials and locations of DES-induced errors.

### Where stimulation is delivered determines when its delivery is maximally disruptive

Having established that both errors and performance speed on correct trials are modulated by physical parameters of DES, we turned to testing the two core predictions made by the analytic framework of causal parametric mapping. The first prediction we tested was that DES will be most effective in disrupting behavior when the timing of its application aligns with the underlying task-relevant computation in the stimulated region. To test this, we computed, for each correct trial with DES, the DES-Picture Onset-Asynchrony: This variable quantifies the timing of stimulation relative to picture onset (Fig. 1). Negative values of DES-Picture Onset-Asynchrony indicate that stimulation was applied before picture onset; positive values indicate that stimulation was applied after picture onset. Trial-level measures of DES-Picture Onset-Asynchrony were normalized to patient-specific average response times on nonstimulated trials. For instance, a −100-ms offset on a given trial in a patient, with an average response time on nonstimulated trials of 1000 ms, corresponds to a DES-Picture Onset-Asynchrony of −10%. This normalization step accounts for the fact that different patients will have different overall levels of performance speed, independent of DES.

**Fig. 3.**
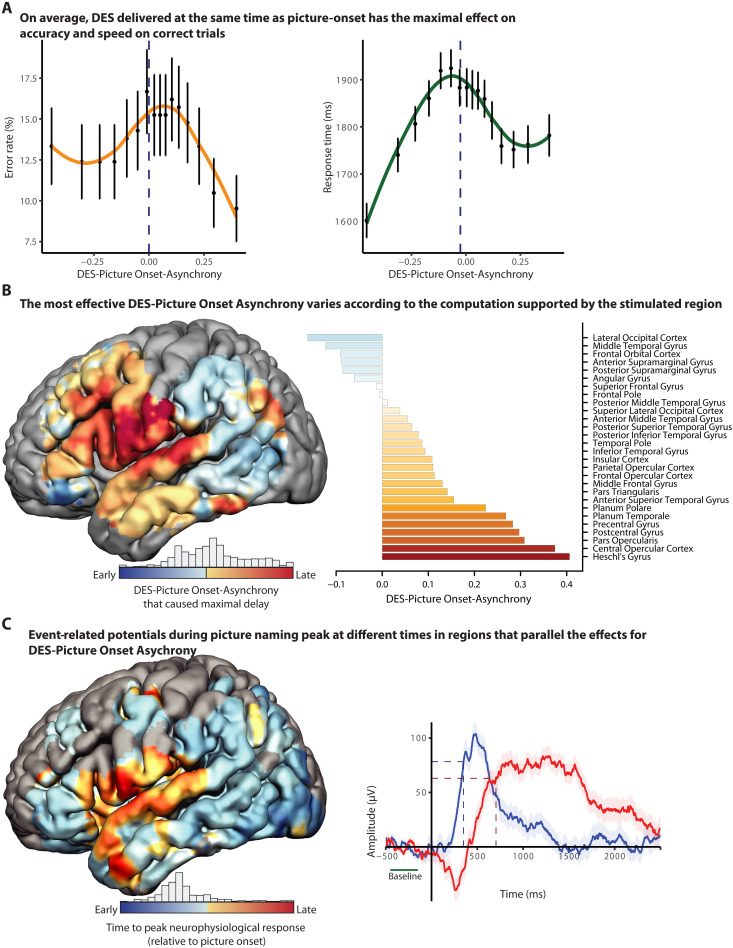
DES-Picture Onset-Asynchrony modulates error rates and correct response times. (**A**) Error rates (orange) and response times (green) varied systematically as a function of DES-Picture Onset-Asynchrony. Both error likelihood and response times peaked when DES was delivered nearly coincident with picture onset (−1 and −4.3%, respectively), supporting the clinical maxim that DES is most effective when delivered coincident with picture onset. (**B**) Sliding window analyses were carried out within a searchlight framework to assess anatomical dissociations in the effect of DES-Picture Onset-Asynchrony on performance. In posterior regions, early DES-Picture Onset-Asynchrony (DES applied early with respect to picture onset) caused the greatest delay in response times. By contrast, in speech sensorimotor regions, relatively later DES-Picture Onset-Asynchrony (DES applied late with respect to picture onset), caused the greatest delay in response times. (**C**) ERP amplitude was measured from a separate cohort of patients with intracranial electrodes implanted for seizure localization. Time-to-peak neurophysiological response values were computed at the level of individual electrodes and then aggregated and mapped within a searchlight framework across cortical surface regions with sufficient coverage. This revealed a systematic anatomical gradient in ERP timing, with warmer colors indicating later peaks relative to picture onset. Traces from two representative electrode contacts illustrate the time-to-peak computation and its normalization to each participant’s baseline response time, parallel with the approach used for DES-Picture Onset-Asynchrony.

We first assessed the relation of error rates and response times to DES-Picture Onset-Asynchrony across the whole dataset, without stratification of trials by anatomic location of DES. That initial analysis showed that the highest probability of error was observed when DES was applied nearly simultaneously with picture onset (Fig. 3A; −1% DES-Picture Onset-Asynchrony). That finding substantiates the long-established practice of aligning DES to the onset of a to-be-named picture (*4*). In parallel to the effect of DES-Picture Onset-Asynchrony on error rates, response times were also maximally slowed when the application of DES coincided with stimulus presentation (Fig. 3A; −4.3%).

The finding that DES-Picture Onset-Asynchrony around 0 is associated with maximal error rates and maximal slowing of response times is based on averaging over anatomically diverse stimulation sites. We thus sought to test whether DES to regions supporting lexical semantic processing will have a more disruptive effect on processing speed when applied early with respect to picture onset, whereas DES to regions supporting phonological and phonoarticulatory planning and production will have a more disruptive effect on processing speed when applied late relative to picture presentation. We used a temporal sliding window analysis within a searchlight framework to identify, for each location in the search space, the DES-Picture Onset-Asynchrony that caused the maximal delay in performance speed (Fig. 3C and movie S1). For posterior lateral temporal and inferior parietal regions, DES maximally slowed correct responses when applied early relative to picture presentation. By contrast, for speech sensorimotor regions, DES maximally slowed correct responses when applied late relative to picture presentation. A notable exception to this pattern was pars orbitalis, where early stimulation maximally slowed performance speed on correct trials. Pars orbitalis is known to be involved in early stages of visual processing of images and has been argued to be a cortical source of top-down input into posterior temporal-occipital regions to facilitate detailed visual processing (*38*). Electrophysiologically, this manifests as an early decrease in gamma power, which precedes the activation of pars triangularis, pars opercularis, and ventral sensorimotor cortex (vSMC) by several hundred milliseconds (*39*).

**Fig. 4.**
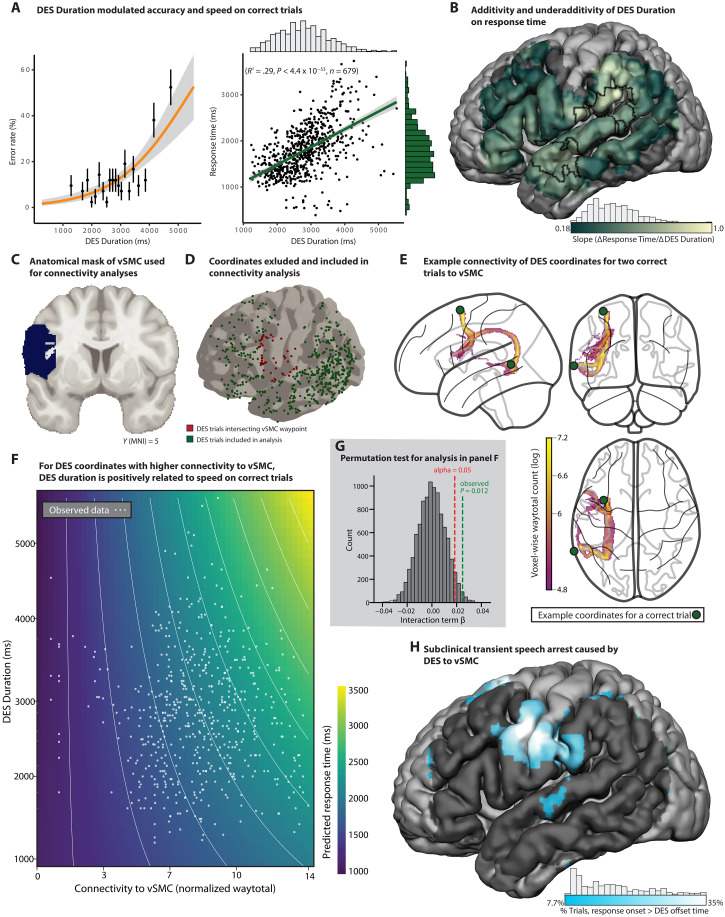
DES Duration identifies functional bottlenecks across processing stages in speech production. (**A**) Logistic regression showed that a longer stimulation duration increased the likelihood of error (OR = 1.0008 [95% CI, 1.0006 to 1.001], *P* < 0.0001; green and orange ticks denote accurate and error trials, respectively). For correct trials (right), increased DES Duration was associated with longer response times [*F*(1,677) = 281.5, *P* < 0.0001]. (**B**) The slope of ∆RT/∆DES Duration is mapped across cortical voxels with significant positive slope (*P* < 0.05), indicating the extent to which each region supports a functional bottleneck. Sites with slopes significantly greater than all other regions (*t* > 1.96; *P* < 0.05) included the vSMC, anterior supramarginal gyrus, and superior temporal gyrus (STG). (**C**) The target vSMC region was anatomically defined using the Harvard-Oxford atlas. (**D**) To ensure independence in connectivity analyses, stimulation sites overlapping the vSMC mask (red) were excluded. (**E**) Example probabilistic tractography from stimulation sites in middle temporal and superior frontal gyri to the vSMC revealed likely pathways along the arcuate fasciculus and frontal aslant tract, respectively. (**F**) A multiple linear regression model demonstrates that connectivity strength significantly modulates the relationship between DES Duration and response time (β = 0.0248, one-tailed *P* < 0.006). The heatmap depicts model-predicted response times across a grid of DES Duration and connectivity values, demonstrating that the effect of DES Duration on response time is amplified at DES sites with stronger connectivity to the vSMC. Observed trial values are overlaid in white for reference. (**G**) The interaction shown in (F) was validated by nonparametric permutation testing (10,000 iterations, *P* < 0.013). (**H**) The anatomical distribution of brain sites where response onset occurred only after DES offset at a rate that exceeded the baseline error rate (7.7%), revealing loci of functional processing blocks. The highest proportion of those trials (~35%) were localized to the vSMC, consistent with long-standing observations of speech arrest resulting from disruption of that final-stage motor planning.

The fact that the anatomic location of DES modulates how DES-Picture Onset-Asynchrony affects performance speed means that DES is not causing a general, nonspecific, slowing in performance regardless of where stimulation is delivered [for discussion, see (*8*, *40*)]. DES is differentially affecting some processes in word production over others, according to the location and timing of where and when DES is applied. This conclusion should not be taken to imply that the effect of DES is confined to the site of stimulation—electrical stimulation current spreads and is differentially propagated down white matter pathways (*36*, *37*, *41*), as demonstrated empirically above (see Figs. 2 and 4). Nonetheless, the separation of temporally early versus late stages of processing, by site and timing of DES delivery, demonstrates that early versus late epochs within semantically driven word retrieval are functionally separable during awake language mapping. These findings generalize a prior observation (*42*) in a single patient that errors were more likely when DES to posterior occipitotemporal regions was applied early, rather than late, relative to picture onset time.

One question that may be raised is whether some of the variability in DES-Picture Onset-Asynchrony across brain regions is driven by surgeon-related biases in when different regions of the brain tend to be stimulated, relative to task performance. To address this, we ran a control analysis that normalized DES-Picture Onset-Asynchrony at each point in the brain. This was done by computing the median DES-Picture Onset-Asynchrony across all accurate trials intersecting each searchlight location and subtracting that median value from the value of DES-Picture Onset-Asynchrony corresponding to the greatest disruption in response time for that searchlight. The interaction between the anatomic location and the time at which DES exerted the largest disruptive effect on response time remained unchanged after this normalization step (fig. S3).

As a final analysis to validate DES-Picture Onset-Asynchrony, we sought convergent evidence from independently measured intracranial neurophysiological data. Intracranial stereoencephalographic (SEEG) recordings were analyzed from a separate cohort of 11 epilepsy patients while they performed a picture-naming task. We tested whether there was alignment, in stereotactic space across the two independent patient groups, between values of DES-Picture Onset-Asynchrony associated with maximal disruptions in performance speed and the timing of peak neurophysiological responses during picture naming, as measured with event-related potentials (ERPs; see movie S2 for the spatiotemporal activation pattern). As shown in Fig. 3E, there was a positive relation between the two independent measures [fig. S4; linear regression: *R*^2^ = 0.281; *F*(1,91) = 35.6, *P* < 0.0001; rank-based: ρ = 0.52, *P* < 0.0001]: Regions of the brain for which DES must be delivered late to maximally disrupt performance speed (positive values of DES-Picture Onset-Asynchrony) overlapped with regions that were active relatively late after picture onset (later time-to-peak neurophysiological responses). In that analysis we chose to use ERPs to quantify the timing of the neurophysiological responses because their temporal resolution is superior, relative to gamma-band activity, which involves temporal smoothing to achieve time-frequency decomposition. Nonetheless, a parallel analysis using gamma-band activity demonstrated the same positive relationship between DES-Picture Onset-Asynchrony and neurophysiological activity [*R*^2^ = 0.164, *F*(1,53) = 10.4, *P* < 0.003]. These findings reinforce the conclusion that the relation of DES-Picture Onset-Asynchrony to performance speed is due to disruption of anatomically dissociable processing stages in the language production system.

### Mapping functional bottlenecks in word production

The second prediction made by causal parametric mapping is that the covariance between the duration of DES and response times on correct trials will index the degree to which the stimulated region represents a functional bottleneck in processing. Building on prior work with dual-task paradigms to map processing stages using response time variance in healthy participants (*24*), we used the slope of the relation between ΔResponse Time and ΔDES Duration to infer the degree to which a given stimulated brain region supports a functional bottleneck. At the limit, disrupting processing at a functional bottleneck for time *t* will lead to a delay of time *t* in response onset time (an additive relation of ΔResponse Time and ΔDES Duration). By contrast, disrupting a region that supports a process that is distributed across multiple other regions will result in a delay on processing that is less than *t* (underadditive relation). This is because anatomically distributed processes are more likely to depend on redundant neuronal systems that can compensate for local disruptions. We tested these predictions first at the level of the entire dataset and then regionally, using searchlight analyses.

Across all trials paired with DES, there were positive relations between DES Duration and both error likelihood {Fig. 4A; odds ratio (OR) = 1.0008 [95% confidence interval (CI), 1.0006 to 1.001], *P* < 0.0001} and response times on correct trials (Fig. 4A; *F*(1,677) = 281.5, *P* < 0.0001). For correct trials, across all stimulated regions, the effect on response time was underadditive: For every 100-ms increase in stimulation duration, there was a 38-ms increase in response time.

Searchlight analyses then measured the slope of the relation between DES Duration and Response Time at each location in the brain with coverage across the DES dataset (estimates of slope at each voxel were thresholded at *P* < 0.05). The analysis identified a set of perisylvian regions that overlapped the regions identified in the accuracy analysis (see Fig. 2D). The highest values for the slope of ΔResponse_Time/ΔDES_Duration approached 1.0, indicating near-perfect additivity. A slope approaching 1.0 was observed in the anterior supramarginal gyrus [Montreal Neurological Institute (MNI) = −65, −36, 29] and posterior vSMC (MNI = −55, −16, 18), which support phonological and phonoarticulatory planning and control, respectively (*30*, *43*, *44*). These findings indicate that regions that support stages of word retrieval at or after phonological encoding represent strict bottlenecks: For every 100-ms increase in DES Duration, there was ~100-ms delay in response times on correct, negative mapping trials (Fig. 4B). By contrast, in regions known to support lexical semantic processing, which are more widely distributed in parallel across both hemispheres, the slope of ΔResponse_Time/ΔDES_Duration was underadditive. For instance, in the left posterior middle temporal gyrus (MNI = −59, −56, 0), for every 100-ms increase in DES Duration, there was a 21-ms increase in response times.

We also tested whether the slope of ΔResponse_Time/ΔDES_Duration varied according to structural connectivity with the vSMC. To maintain independence, the vSMC was defined anatomically using the Harvard-Oxford atlas (*45*), and any DES coordinates that intersected the vSMC were excluded from analysis (*n* = 53; Fig. 4, C to E). For the remaining trials, we computed subject-specific probabilistic tractography from each DES coordinate to the vSMC. A multiple linear regression model predicted response time with the independent variables of stimulation duration, connectivity strength, and their interaction. The interaction term was significant (Fig. 4F; β_interaction = 0.0248, one-tailed *P* < 0.006), indicating that higher connectivity to the vSMC modulates the effect of stimulation duration on response time. The robustness of this finding was further confirmed using a non-parametric permutation test (10,000 iterations), which indicated the interaction effect exceeded the 95th percentile of the null distribution (Fig. 4G; *P* < 0.012; threshold = 0.0185). These findings indicate that anatomic connectivity to the speech sensorimotor system increases the likelihood that DES causes additive delays. This supports the assumption (see Fig. 2) that DES propagates down white matter pathways and that the vSMC functions as a strict bottleneck in speech production.

### Transient speech arrest caused by DES to the last stage of cortical speech processing

As noted above, all analyses of response time effects reported to this point were based on the 93% (693/745) of correct DES trials for which the spoken target response initiated before the end of the DES train. Trials on which patients were correct, but for which initiation of the response did not begin until after discontinuation of DES, were excluded from all analyses of responses times above. This was to not “contaminate” the principal response time analyses with trials that might represent subclinical transient speech arrest [note (see Materials and Methods) that all hesitation errors were excluded from analyses of response times]. If those 7% of trials represent a form of subclinical transient speech arrest, they should be anatomically concentrated in regions that support the final stages of cortical speech processing. In line with that expectation, a searchlight analysis identified the vSMC (Fig. 4C) as being the only region in the dataset where DES was associated with a significantly elevated likelihood of patients not being able to initiate the target response until after the stimulation train ended (thresholded at the baseline error rate: >7.7%). At the peak location in the speech sensorimotor cortex, 35% of correct responses did not initiate until after discontinuation of DES. These findings are in line with the long-standing clinical observation that DES on both sides of the central sulcus in the vSMC can cause speech arrest (*2*, *43*), potentially through disruption of phonoarticulatory and anticipatory sensory feedback within a forward model of production (*44*), spreading activity to motor regions, or both. More broadly, these findings further underline the tight coupling between response times on correct, or so-called negative mapping trials, and overt errors.

### Predicting response times on negative mapping trials

**Fig. 5.**
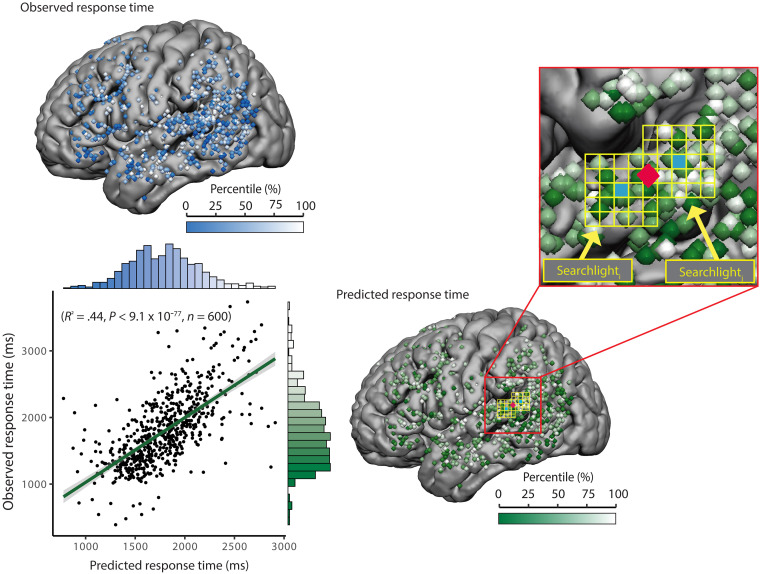
Predicting response times on negative mapping trials. A linear multiple regression model with predictors DES Duration, DES-Picture Onset-Asynchrony, and DES amplitude (in mA) was fit at each searchlight to *n*−1 trials and used to predict the response time on the *n*th trial. That model accounted for 54% of the variance in response times [*R*^2^ = 0.542; *F*(1,389) = 460.9; *P* < 0.0001; see text]. Anatomical distribution of real and predicted response times on correct trials plotted on template brains, with the corresponding color bars (above and right, respectively). The scatterplot shows real versus predicted data for a simpler regression model that did not include DES amplitude and accounted for 44% of the variance in the observed response times on correct negative mapping trials [*R*^2^ = 0.438; *F*(1,598) = 465.3; *P* < 0.0001; see the Supplementary Materials for extended analyses].

We have shown that stimulation amplitude, DES-Picture Onset-Asynchrony, DES Duration, and the location of DES account for previously unexplored and unexplained variance in response times on negative mapping trials. To quantify the generalizability of causal parametric mapping, we performed a series of leave-one-out cross-validation analyses on response time data from correct trials (see the Supplementary Materials for full details). A linear model was fit to *n*−1 trials at each searchlight location in the brain, with factors DES Duration, DES-Picture Onset-Asynchrony, and DES amplitude (mA). That fitted model was used to predict the left-out trial at that searchlight location. This was iterated over all trials at that searchlight location and over all searchlight locations (fig. S6). Across all accurate trials that had sufficient coverage (*n* = 605), DES Duration, on its own, explained 38.4% of the variance in response times [*F*(1,599) = 373.2; *P* < 0.0001]. DES-Picture Onset-Asynchrony, on its own, predicted 7.3% of the variance in response times [*F*(1,595) = 46.8; *P* < 0.0001]. A multiple linear regression with both DES Duration and DES-Picture Onset-Asynchrony accounted for 43.8% of the variance in response time [*F*(1,598) = 465.3; *P* < 0.0001; Fig. 5A], whereas adding DES amplitude to the regression model explained an additional 6.8% of the variance [*R*^2^ = 0.542; *F*(1,389) = 460.9; *P* < 0.0001]. This predictive framework demonstrates that ~50% of the response time variance from negative mapping trials carries information about underlying language organization in the brain. Additional supporting analyses (Supplementary Materials) establish the importance of using region-specific predictive models—equivalent models that do not account for the location where DES was administered do not achieve the same level of performance.

Our goal here has been to demonstrate that response times on negative mapping trials are not stochastic noise but rather represent a functionally informative readout of language organization. The success of this approach motivates future studies that could productively model the full range of factors known to affect response times, such as picture familiarity, lexical frequency, and item repetition, to maximize the variance that can be explained on correct response times.

## DISCUSSION

Since its inception half a century ago, functional language mapping with DES has been used for clinical purposes in only one way: as a binary readout of patient performance. Brain regions are classified as “eloquent” (language positive) if errors are elicited by DES or “safe to remove” (language negative) if no errors are elicited by DES. Clinically, that “all-or-none” approach for delineating functional boundaries has resulted in an efficient and rapid intraoperative process to stratify the neurocognitive risk of resecting a given volume of tissue. Scientifically, the “all-or-none” approach was aligned with now classic views of the neurobiology of language that emphasized functionally separable cognitive processes localized to a restricted set of core language centers. However, research over the past two decades has demonstrated many instances in which well-defined functional processes are widely distributed across networks of interconnected regions (*46*–*49*). Even the relatively ballistic task of spoken picture naming involves parallel information streams that map lexical semantic representations to phonoarticulatory systems (*34*, *50*, *51*). Nonetheless, DES as a technique has remained largely unchanged in clinical practice over the entirety of its history. Our goal here has been to test whether continuous measures of performance are meaningfully related to continuous physical parameters of DES.

The conceptual advance of causal parametric mapping is that it identifies functionally separable processing stages—rather than merely “detecting” where language function is present. This holds the potential, through further clinical validation as discussed below, to shift the proximate goal during awake mapping from determining whether a region is eloquent to testing a hypothesis about what the region does. The closest analog to this approach is qualitative analysis of overt errors generated during DES language mapping (*12*, *52*–*54*). When overt errors are produced, the type of error can be powerful in constraining the processes that were disrupted by DES (regardless of whether those processes are local or not with respect to the site of stimulation). For instance, stimulation of speech motor regions can lead to dysarthric responses /chat/ instead of /kat/, whereas stimulation to temporal lobe lexical semantic regions can result in semantic paraphasias [/dog/ instead of /cat/ (*55*)]. However, overt errors are exceedingly rare [~1 to 3% of all DES trials; see (*52*)] and are thus neither a reliable nor a practical measure with which to derive inferences about what processing stage(s) are affected by DES. The most common type of error in the setting of DES mapping is a failure to respond (*12*, *56*), which does not support an unambiguous inference as to the processing stage affected by DES.

Here, we have focused on visual picture naming because it is the most widely used task to map language with DES. Pairing the most appropriate language task with the region being stimulated is critical so that the appropriate neurocognitive processes are engaged when DES is applied to the brain [for review, see (*57*)]. Causal parametric mapping can be extended to any task or domain paired with DES, such as motor function (*58*), math ability (*59*), music processing (*60*, *61*), visual perception (*62*), or other specialized cognitive functions (*57*), including additional language tasks such as sentence production or comprehension (*63*–*66*).

### The tight coupling of clinician and patient behavior

Variability in stimulation timing, duration, and amplitude is inherent to the clinical procedure of DES mapping during awake surgery. This study leveraged variability in those parameters in support of this first proof-of-principle demonstration of causal parametric mapping. However, the fact that clinician behavior is yoked to patient behavior raises important questions about the directionality of causation between clinician behavior and patient behavior. For instance, standard clinical practice is for the surgeon to stimulate the brain until several seconds elapse, a patient makes an error, or a patient starts to produce the correct response—typically, whichever comes first. This could lead to longer response times on correct responses being associated with longer stimulation events, not because of an effect of DES Duration on behavior but because surgeons are “waiting” for a correct response to discontinue stimulation. To address this ambiguity, we tested whether covariance between DES Duration and response time is a result of clinician behavior, in three ways. First, if clinician behavior was driving the covariance structure in the data, the relation would not exhibit regional specificity. As shown in Fig. 4B, whereas the slope ∆Response Time/∆DES Duration is 0.38 across the whole brain, it is ~1.0 in speech sensorimotor and in the anterior supramarginal gyrus. Regional variability of the slope of ∆Response Time/∆DES Duration indicates that the effect is modulated by the underlying functional processes supported by anatomically separable regions. Second, the slope of ∆Response Time/∆DES Duration is higher in regions exhibiting significantly increased error rates (Fig. 2D), compared to all other regions. A formal test showed that the interaction (comparison of slopes) was statistically significant, comparing regions exhibiting a significantly elevated error rate versus regions for which the error rate was not greater than nonstimulated baseline rates (*b* = 0.17, SE = 0.06, *P* < 0.007). Third, the same region for which ∆Response Time/∆DES Duration was the highest, namely, the speech sensorimotor cortex (Fig. 4B), also exhibits the highest likelihood that patients did not respond until after DES was discontinued (Fig. 4C). Thus, on a substantial proportion of trials in the speech sensorimotor cortex (~35%), the surgeon stops stimulating before the onset of the patient’s correct response. Those additional observations in the data indicate that the core finding of covariance between stimulation duration and response time is not driven solely by clinicians adapting their process to patient behavior. Nonetheless, future work within causal parametric mapping must carefully investigate the tight coupling between clinician and patient behavior.

### Potential sources of nonlinearity in causal parametric language mapping

We have developed causal parametric DES mapping in its simplest form by assuming linear relations among the physical parameters of DES and error rates and response times. Furthermore, “dual task” logic makes assumptions about the sequentiality of processing stages. For scientific purposes, it can be productive to start with linear models and reject assumptions of linearity as evidence warrants. Thus, our goal was to maintain the simplest possible set of assumptions going into the analysis—on the expectation that future work, with larger datasets, may be able to achieve higher variance explained by moving beyond simple linear models. There are at least five reasons to anticipate nonlinearities in the mapping of DES parameters to performance.

One of the more frequent types of errors elicited by DES are “hesitations”—the patient ultimately produces the correct response but is delayed and the response is categorized as a hesitation error. Those trials were excluded from analyses of response times for the purposes of this investigation; we further “cleaned” negative mapping response times of values greater than 3.0 SDs from the mean and excluded responses that did not initiate until after discontinuation of DES. The implication is that all analyses of response times herein are based on the central distribution of the data and are not driven by statistical outliers, which might be considered to be subtle (and potentially missed) hesitation errors. Nonetheless, our findings indicate that there is a continuum in performance—from predictable millisecond variation on correct trials, to not being able to speak until after DES is discontinued, to hesitations, and to failures to respond. Future work may consider modeling hesitation errors and failures to respond as supraadditive effects of DES on processing time: DES of duration *t* causes a slowdown in response time that is longer than *t*.

A second source of nonlinearities between temporal parameters of DES and patient performance is interactivity and feedback among the processing stages involved in visual naming (*27*, *31*). The perisylvian language areas that support picture naming involve lexical semantic access and selection, phonological encoding, and phonoarticulatory and laryngeal planning and production. That series of stages is “globally serial” with substantial local interactivity and overlapping temporal windows among adjacent stages of processing (*67*). For instance, aspects of phonological encoding may be engaged before the completion of lexical semantic processing (*27*, *50*). We note that, if anything, interactivity between phonoarticulatory and lexical semantic processing stages works against observing the findings we have reported as we found nearly perfect additive covariance between DES Duration and response time in the vSMC and the anterior supramarginal gyrus.

A third potential source of nonlinearities in causal parametric mapping is due to the fact that DES affects processing not only in the stimulated region but also in structurally and functionally connected regions (*37*, *56*) (see Fig. 4). The effect of DES on behavior is thus due to disruption of processing, both local to the site of stimulation, and nonlocal via propagation of current through functional networks. DES to hubs within functional networks (*68*, *69*) or DES at higher stimulation amperages, which increases current spread (*35*), increases the incidence of errors (*13*) (see Fig. 2C). As a first step toward capturing those effects, we reasoned that proximity of DES on correct trials to the locations of DES-induced errors should modulate performance speed. This analysis was carried out within-subject (each correct trial with DES related to the nearest error in that subject) and between-subject (each correct trial with DES related to the nearest error in any other subject). Proximity was implemented both in terms of Euclidean distance in the brain and connectivity-based distance (probabilistic white matter tractography). We found that, although both Euclidian and connectivity-based measures of distance independently explained performance speed in the between-subject analysis, there was no effect of Euclidean distance over and above the effect of connectivity in the within-subject analysis. Future extensions of causal parametric mapping that integrate intracranial recordings—capturing both local feedback dynamics and distal propagation of current spread—could provide the spatiotemporal precision needed to directly relate neural perturbation dynamics to behavior across the speech production network. More broadly, the fact that temporal parameters of DES modulate performance across perisylvian regions (Figs. 3B and 4B) indicates, minimally, that stimulation affects subnetworks of regions within broader language processing networks.

A fourth source of nonlinearities in causal parametric mapping is the potentially bivalent effect that DES can have on behavior. We have focused on modeling DES as a transient block on, or disruption to, processing. This is a standard interpretation of DES and draws on the notion that stimulation manifests as a transient and reversible lesion at the site it is applied (*70*). However, it has also been demonstrated that intracranial and extracranial stimulation in specific contexts can facilitate performance (*15*, *71*). Whether stimulation inhibits, enhances, or has no effect on performance likely depends, in part, on the state of the system at the time that stimulation is applied (*71*). Thus, representing DES as a transient block on processing may not capture the full range of potential effects that DES could have on processing. If DES is modeled as the introduction of noise to the system, that noise input could occur when computations are underway that support performance, in which case DES would interfere with performance. However, if DES were applied (and removed) to a region before underlying computations are underway, potentiation of local activity may facilitate processing and thereby speed performance.

A fifth source of nonlinearities is that the functional organization of the brain is characterized by both local and global discontinuities. Adjacent structures may be functionally distinct, whereas distal structures may be functionally similar, due to high connectivity. In addition, current spread falls off nonlinearly within a volume of tissue and is differentially amplified down white matter pathways. Thus, there will be nonlinear relations between the Euclidean or connectivity-based distance of a point of stimulation to the nearest location where an error was induced and the effect of that stimulation on behavior.

### Evaluating the clinical significance of causal parametric language mapping

It is important to note that a limitation of the current project is that it was based on the use of nonstandard naming protocols. In awake mapping, the field has been striving to achieve convergence across surgeons and sites in terms of the tests and specific materials that are used for language mapping. Our hope is that this study is part of a broader drive toward convergence and standardization in terms of the tools that are used for language mapping during awake surgery. In support of those goals, we have made the experimental materials used herein available to the community (see Data, code, and materials availability for details).

Current practice using DES to classify brain regions as eloquent versus noneloquent has proven enormously effective in optimizing patient outcomes, in terms of postoperative neurocognitive function, quality of life, and postoperative survival (*5*, *7*, *72*, *73*). Critically, the distinction between DES-induced errors and correct performance will, and we believe always should, hold priority in driving real-time clinical decisions about which brain regions support language function. There can simply be no stronger evidence that a region is critical for task performance than the induction of errors by DES. At the other end of the extreme, some (or perhaps many) negative mapping trials do not carry information that is informative about underlying function. It should not be anticipated that all negative mapping trials contain information about underlying neurocognitive organization. Certainly, for any task, there will be brain regions that, when stimulated, have absolutely no effect on task performance (errors or performance speed). Causal parametric mapping offers a framework to sort out which negative mapping trials do and do not carry important information and how physical parameters of DES can be optimized to maximize its sensitivity to map function.

It remains to be demonstrated whether causal parametric mapping confers a benefit to the clinical process and, ultimately, to patient outcome. Clinically, the proximate goal of awake language mapping is to find the boundaries of functional language areas; the ultimate goal of awake language mapping is to optimize postoperative patient outcome. Optimizing outcome does not only mean avoiding new postoperative neurocognitive deficits. Some postoperative deficits are recoverable and thus do not need to be avoided. Furthermore, in some situations, the cost of incurring postoperative deficits that are unlikely to recover is outweighed by the benefit of an aggressive surgical management of the primary disease.

Clinical validation of causal parametric mapping will require large, multisite, prospectively enrolled cohorts of patients. A future trial could test whether postoperative language outcomes are different based on resection plans that do or do not incorporate adjutant measures from causal parametric mapping, compared to traditional DES-based definition of tissue as eloquent or not eloquent. For instance, future investigations may integrate error rates and performance speed into a single measure for each stimulated brain region. A measure of functional involvement of a given brain site that integrates error rates and performance speed can be tested for how it predicts postoperative neurocognitive function, quality of life, and, for more malignant brain tumors, survival.

Another potential application is to test which putative “language negative” regions in a given patient’s brain do and do not show modulatory effects on performance speed by physical parameters of DES. For instance, it would be valuable to test whether causal parametric mapping allows for shorter mapping sessions while not sacrificing accuracy of functional borders. More broadly, such future clinical work can test whether causal parametric mapping can support real-time inferences about the spatiotemporal architecture of processing stages in speech production.

Causal parametric mapping provides a framework to maximize the sensitivity of DES to cause errors. This means that causal parametric mapping may be effective for minimizing false-negative trials, thereby maximizing the sensitivity of DES to map function in the traditional manner through elicitation of errors. Granular understanding of how to maximize the sensitivity of DES, while not compromising on its specificity to map isolated neurocognitive processes, has implications for assessing the sensitivity and specificity of other brain mapping techniques, which are commonly benchmarked against DES mapping (*6*, *9*, *56*, *74*, *75*).

## MATERIALS AND METHODS

### Awake neurosurgery patient participant recruitment

Neurosurgical patients scheduled for awake craniotomy were recruited between 2014 and 2023 from two institutions: the University of Rochester Medical Center (URMC) and the University of Pittsburgh Medical Center (UPMC). Patients were enrolled in a larger prospective study on cognitive brain mapping in neurosurgical patients, conducted by the Program for Translational Brain Mapping at the URMC and Carnegie Mellon University (*76*, *77*). For the current analysis, a subset of 19 patients was selected from the larger cohort based on the following criteria: (i) successful completion of awake DES language mapping during surgery, (ii) the presence of a left-hemisphere lesion, and (iii) native English speakers (see table S1 for details). All participants provided informed consent under protocols approved by the University of Rochester Research Subjects Review Board (URMC RSRB: STUDY30837 and STUDY0206) and by the University of Pittsburgh Institutional Review Board (UPMC IRB: STUDY20110340), with reliance agreements from the Carnegie Mellon University (CMU reliance on URMC: STUDY2019_00000077; CMU reliance on University of Pittsburgh IRB: STUDY2021_00000391). As specified in those single institutional review board (sIRB) protocols, all tasks that patients completed during surgery were at the request of the clinical team and for proximate clinical purposes. These protocols permitted the research team to be present during standard of care neurosurgical procedures and to prospectively collect clinical data in support of scientific aims. Informed consent to share patient voice recordings, with faces blurred, was obtained separately for individuals who appear in movie S1. Both individuals shown in movie S1 were patients of the URMC and thus completed (or a representative for the patient completed) a standard URMC Health Insurance Portability and Accountability Act (HIPAA) release.

### Neurosurgical procedure and intraoperative mapping

Because of the proximity of lesions to suspected eloquent motor and/or language cortex, patients underwent tumor or epileptogenic zone (EZ) resections using an awake procedure with language and sometimes motor mapping (*72*, *78*, *79*). This approach followed established protocols at the URMC and the UPMC [for prior studies using this protocol, see (*30*, *67*, *75*); for an overview, see (*70*)]. Given that all lesions were located in the left hemisphere, patients were positioned on the operating table on their right side, with their head secured in a Mayfield head-holder. This positioning allowed for both optimal surgical access and for patients to see a small monitor that was positioned directly in their line of sight. A video camera recorded patient’s faces, and a directional microphone recorded all responses, time locked to stimulus presentation.

Following a craniotomy tailored to each patient’s lesion, and after exposing the cortical surface, patients were gradually brought to wakefulness to participate in awake intraoperative mapping. Once awake, patients performed a picture-naming task, during which DES was applied on approximately one-third of the trials (see table S2 for details), always at the surgeons’ discretion. Patient responses were captured using a directional microphone, projected through a speaker system for the clinical team, and recorded for later analysis. The primary clinical goal of the awake task was to provide the neurosurgical team with real-time causal evidence regarding the boundaries of eloquent cortical regions (both language and motor areas), allowing for the identification of critical tissue to preserve during resection. DES was administered using a bipolar Ojemann stimulator (Nicolet), with stimulation amplitude set at 0.5 mA below the after-discharge threshold for the stimulated region. Across all mapping sessions, stimulation amplitudes ranged from 1 to 9 mA (table S2).

### Registration of intraoperative stimulation sites to preoperative MRI

The location for each site of intraoperative DES was acquired in real time using the Brainlab system for cranial navigation and confirmed postoperatively through video verification blind to behavioral performance. Those coordinates were subsequently analyzed offline by projecting them into native T1 anatomical space and subsequently to the normalized MNI space. When subject-specific neuronavigation coordinates were not available for a given stimulation event, coordinates were drawn on a three-dimensional (3D) rendering of that patient’s preoperative high-resolution MR images by author J.R.B. and then verified by author A.N.M. (7 years of neurosurgical experience) using BrainVoyager software (version 22.2). This process was always blind to trial level behavioral effects of stimulation (by keeping the audio channel off during DES localization). For all stimulation events, coordinates in native space were extracted, and then a spatial transformation of preoperative MR images into the standard MNI 152 space was performed using nonlinear registration (SPM12; https://fil.ion.ucl.ac.uk/spm/software/spm12/). A 5-mm-radius sphere was defined around each stimulation coordinate to represent each stimulation event [for details and precedent, see (*68*)]. After MNI normalization, the location of coordinates were again confirmed to ensure they remained on the cortical surface.

### Scoring of intraoperative performance

During the intraoperative mapping procedure, patients were presented with stimuli to be named using in-house software (previously named StrongView, now MindTrace Measure). Established clinical practice (*1*, *4*, *7*, *11*) is for the surgeon to apply DES to the brain coincident with the onset of an experimental stimulus that must be named. Most patients read a written preamble (e.g., “Here is a”) before naming the target noun (*n* = 16); the remaining patients produced only the target noun (bare noun naming; *n* = 3). Those choices were at the discretion of the clinical team directing the mapping, based on clinical goals. The timing between trials varied between 3 and 7 s, tailored to each patient’s natural pace, also determined by the clinical team (table S2). Some mapping sessions included trials with written word and Arabic numerals to be read: Analyses reported in herein focused on only picture-naming trials. Patients completed anywhere from 35 to 274 trials, depending on the length of the mapping session. A total of 2498 intraoperative picture-naming trials were recorded across the 19 patients. A total of 262 trials were excluded from analysis due to interruptions unrelated to stimulation (e.g., patient talking with clinicians). Of the remaining 2236 trials, 867 were paired with DES to the left hemisphere. Trials were considered to be paired with DES if the stimulating probe was in contact with the cortical surface during the period between when the stimulus was presented to the patient, and the patient initiated a spoken production of the target response. The remaining nonstimulated trials (*n* = 1439) served as a baseline comparison to control for general intraoperative factors (e.g., semilateral positioning, recent sedation, and medications)

Four types of responses were scored as errors and removed from analyses of response times: (i) hesitations, as noted by the clinical team during mapping; (ii) failures to respond for the duration of the trial; (iii) production of incorrect names; and (d) verbal disfluencies (stuttering, utterance repairs). Response onset times on correct trials were measured as the time between onset of the visual picture and onset of the spoken production of the target noun. Response time and video analyses were conducted offline using the open-source software Audacity (version 3.6.3) and Adobe Premiere Pro (version 22-24.63). Furthermore, and to ensure that any potential hesitation errors were removed from analyses of response times on correct trials, including potential hesitations that were missed by the clinical team in the moment during mapping, correct response times greater than 3 SDs from the mean (1.5%; 11/745) were excluded from analyses of response time variance. Correct trials in which patients initiated their spoken response after the offset of the DES train were also excluded from primary response time analyses (7%; 52/745) as these trials may reflect transient disruptions that resolved before response onset. These trials were retained for accuracy analyses and examined separately (see Fig. 4).

### Statistical analyses of intraoperative data

All primary statistical analyses were performed using R (version 4.3.2; R Foundation for Statistical Computing, Vienna, Austria) with RStudio (Posit Software, Boston, MA) and Python. Graphs were produced using the ggplot2 package and matplotlib. Statistical tests included two-sample *t* tests, linear regression, and chi-square tests. For analyses related to DES timing (DES-Picture Onset-Asynchrony, DES Duration), we used a sliding window analysis to detect effects on accuracy and response time. Sliding window analyses were performed with a window size covering 25% of the total data and a step size of 5%, resulting in an 80% overlap among consecutive windows. DES offset-related effects were modeled through both the inclusion of stimulation duration (DES offset = DES onset + DES Duration) and though a separate examination of trials in which responses began only after discontinuation (i.e., offset) of stimulation.

To ensure that analyses of DES-Picture Onset-Asynchrony and DES Duration were not driven by outliers, the distributions of both variables were cleaned of values 3 SDs beyond the mean, in both positive and negative directions (25/867 trials; 2.8%). To account for different processing speeds among patients, the DES-Picture Onset-Asynchrony for each DES event was normalized to the average response time for that respective patient’s nonstimulated trials. Regional analyses to assess local stimulation effects were conducted using a searchlight approach, using in-house scripts written in Matlab with BVQX tools (now named NeuroElf; https://neuroelf.net/). Whole-brain searchlight maps were generated using a mask aligned with the MNI 152 template space. For each voxel (central voxel), the “searchlight” analysis evaluated a 5 by 5 by 5 voxel cube (*n* = 125 voxels) surrounding the central voxel in the MNI space. Analyses were performed for all naming trials where a DES event (within a 5-mm radius) intersected the searchlight. A minimum of 10 naming trials per searchlight was required for statistical analysis, and the results from each statistical model were assigned to the center voxel of the cube.

### MRI parameters

Whole-brain MRI was conducted on a 3-T Siemens MAGNETOM Trio scanner with a 32- or 64-channel head coil at the University of Rochester or on a 3-T Siemens Prisma scanner at the University of Pittsburgh. High-resolution structural T1-weighted images acquired at the University of Rochester (*n* = 16) used a magnetization-prepared rapid acquisition gradient echo (MPRAGE) sequence [repetition time (TR) = 2530 ms, echo time (TE) = 3.44 ms, flip angle = 7°, field of view (FOV) = 256 mm, matrix = 256 by 256, 192 sagittal slices, and voxel size = 1 mm by 1 mm by 1 mm]. For participants scanned at the University of Pittsburgh (*n* = 3), structural T1-weighted images were also acquired using a 3D MPRAGE sequence (TR = 2300 ms, TE = 1.99 ms, inversion time = 900 ms, flip angle = 9°, FOV = 256 mm by 256 mm, matrix = 256 by 256, and voxel size = 1 mm isotropic). Parallel imaging was used (GRAPPA, acceleration factor *R* = 2), and whole-brain coverage was obtained in the sagittal plane.

### Diffusion MRI acquisition

Diffusion-weighted imaging (DWI) data were acquired using three protocols across 17 of the 19 participants: (i) single-shell DWI with gradient-recalled echo (GRE) field maps, (ii) single-shell DWI with reverse phase-encoding pairs (e.g., AP/PA), and (iii) multishell high-angular resolution diffusion imaging (HARDI; up to *b* = 4000 s/mm^2^). For the first 15 participants (protocols 1 and 2), diffusion MRI was acquired using a single-shot spin-echo echo-planar imaging (EPI) sequence [60 directions at *b* = 1000 s/mm^2^ and 10 *b* = 0 volumes; TR = 8900 ms, TE = 86 ms, FOV = 256 mm by 256 mm, matrix = 128 by 128, voxel size = 2 mm by 2 mm by 2 mm, and 70 axial slices; anterior-to-posterior (AP) phase encoding]. A double-echo gradient echo field map (ΔTE = 2.46 ms; EPI dwell time = 0.75 ms) with matching resolution was acquired to correct for *B*_0_ inhomogeneities. For the final two participants, HARDI data were acquired on the Siemens Prisma scanner using a single-shot spin-echo EPI sequence with multiband acceleration (factor = 4). A total of 258 volumes were collected, including 251 diffusion-weighted directions (up to *b* = 4000 s/mm^2^) and 7 *b* = 0 s/mm^2^ volumes. Imaging parameters were as follows: TR = 2500 ms, TE = 99.6 ms, flip angle = 90°, FOV = 256 mm by 256 mm, matrix = 128 by 128, and voxel size = 2 mm isotropic. Data were acquired with posterior-to-anterior (PA) phase encoding, with a total readout time of 71.4 ms and an effective echo spacing of 0.68 ms.

### Diffusion MRI preprocessing

Diffusion data were preprocessed using tools from the FMRIB Software Library [FSL v6.0.6 or later; (*80*)]. Brain extraction was performed on all diffusion and T1-weighted volumes using FSL’s BET (*81*). Preprocessing pipelines were adapted to account for variation in acquisition protocols, including distortion correction and coregistration to anatomical space. For datasets with reverse phase-encoding pairs (AP/PA), susceptibility-induced distortions were corrected using FSL’s topup and eddy (*82*) tools with subject-specific acquisition parameter files. Eddy current correction included motion modeling and outlier replacement. For datasets with GRE field maps, distortion correction was performed using FSL’s fugue tool following preparation with fsl_prepare_fieldmap. A 3D Gaussian smoothing kernel (σ = 4 mm) was applied to improve spatial regularity. For multishell HARDI datasets, *b* = 3500 s/mm^2^ volumes were isolated using fslselectvols. Phase-encoding reversal data were available for both participants, allowing full topup and eddy correction. T1-weighted images were registered to diffusion space using FSL’s linear registration tool [flirt; (*83*)]. After preprocessing, all datasets were converted into compatible formats for modeling with bedpost and bedpost_gpu, which use Bayesian modeling to estimate the posterior distributions of fiber orientations using a two-fiber model and automatic diffusivity estimation.

### Probabilistic tractography

Structural connectivity was estimated using probtrackx2 or probtrackx2_gpu, which samples from the posterior distributions by bedpost (*83*, *84*) to generate streamlines, For each stimulation site, a 5-mm-radius spherical seed mask was generated and transformed to subject-specific diffusion space using std2imgcoord (fig. S2). For the network proximity to errors analysis, 5000 streamlines were propagated per voxel. For vSMC-specific connectivity analyses, 10,000 streamlines per voxel were used. Target masks—including individual error DES coordinates and anatomically defined vSMC mask—were registered to individual diffusion space using stdimgcoord and flirt, respectively. A midline sagittal slice was used as an exclusion mask to avoid fibers that cross into the right hemisphere. Tracking was performed with a curvature threshold of 0.2 and a step length of 0.5 mm. Using FSL’s network mode option, only streamlines that originated at the seed and passed through the target mask were kept. Connectivity strength was quantified as the number of streamlines reaching the target (i.e., waytotal count) and log-transformed before statistical analysis to reduce skew. ROI (region of interest) masks were visually inspected and refined as needed (e.g., medial shifting or boundary cropping) to avoid overlap with stimulation sites or artifacts. All preprocessing and tractography steps were implemented using containerized FSL pipelines and custom bash/Python scripts on a GPU-accelerated SLURM cluster.

### SEEG patient participant recruitment and testing

Data from 14 patient participants who were recruited and tested during their phase II SEEG monitoring for epilepsy at the UPMC Epilepsy Monitoring Unit (EMU) between June 2022 and March 2023 were included. All patients had previously undergone phase I in-patient evaluation with scalp EEG. SEEG trajectories for implanted electrodes were based on working clinical hypotheses based on the potential anatomical location and extent of the EZ, as well as the possible locations of relevant functional cortical areas in each patient. After obtaining informed consent, the patients were enrolled in the study (see table S3 for patient details).

All patient participants completed a visually cued naming task. A computer monitor was fixed to a desk adjacent to the patient’s bed and placed directly in the patient’s line of sight as they were seated in the hospital bed. Stimuli were presented using in-house software (StrongView) every 3 s, with an intertrial interval (ITI) of 500 ms. A fixation cross was presented at the center of the screen during the ITI. Visual stimuli were line drawings, images, words, and numbers; line drawings and images that were correctly named were included in analysis for the present study. Patients were instructed to verbally respond to each stimulus as quickly and accurately as possible. Responses were recorded using a directional microphone for offline analysis. Accuracy and response times were scored using open-source Audacity software by author J.R.B. Two subjects were excluded from analysis due to technical errors in audio recording of responses, and one was excluded due to a lack of contacts implanted in the left hemisphere.

### SEEG electrode reconstruction, selection, and signal preprocessing

Data acquisition of SEEG recordings was performed with a 256-channel Natus clinical system sampled at 1024 or 2048 Hz. Signals from all channels were referenced to common monopolar contacts placed on the scalp. Each patient had a post–SEEG implantation high-resolution Computed Tomography (CT), which was registered to their preoperative high-resolution T1 using Brainstorm (*85*). Spatial transformation of preoperative MR images into the standard MNI 152 space was performed using nonlinear registration (SPM12). Standard SEEG preprocessing techniques were conducted in Matlab using fieldtrip (*86*) and involved application of a 0.1- to 200-Hz band-pass finite impulse response (FIR) filter, notch filters at 60/120/180 Hz, and downsampling to 1024 Hz (when necessary). A total of 1380 picture-naming trials were recorded across all patients (excluding 256 trials on which patients made errors). Trials were epoched relative to stimulus onset (−500 to 2500 ms), and baseline correction was applied by subtracting the mean voltage of the prestimulus period (−450 to −150 ms) from each trial. The resulting baseline-corrected local field potential (LFP) voltage traces were then averaged across trials to compute the time-domain ERP waveforms for each contact.

High-gamma activity was extracted by applying a two-way zero-phase FIR band-pass filter (70 to 150 Hz), followed by Hilbert transformation to compute the analytic signal. The resulting amplitude envelope was smoothed using a third-order Savitzky-Golay filter with an 11-point window. For each trial, the smoothed gamma envelope was normalized by subtracting the mean amplitude within a fixed baseline window (150 to 450 ms poststimulus onset) to isolate event-related high-gamma responses. This baseline-corrected gamma signal was then averaged across trials to yield trial-averaged high-gamma time courses. Principal analyses (Fig. 3) and electrode selection (described below) were based on LFPs; all effects remained when using high-gamma power (see text).

A total of 1822 contacts were implanted in the left hemisphere across all patients (fig. S5). Contacts that did not intersect brain parenchyma were excluded from all analysis (*n* = 302; 16.6%). To assess whether a contact was responsive to the task, we used the strict criterion of whether its activity during naming was significantly different from baseline for at least 300 ms. We defined the onset of time windows with 50-ms steps, starting from stimulus onset and extending to 1500 ms poststimulus. A nonparametric cluster-based permutation test, using the Monte Carlo method with 1000 permutations, was applied to determine the null distribution against which to compare activation during naming (baseline window: −450 to −150 ms), using a two-tailed dependent samples *t* test (α = 0.05). Contacts that did not show a significant difference from baseline on ERP analysis for at least 300 ms during correct naming trials were excluded from further analysis (*n* = 830, 54.6%).

Six hundred and ninety contacts (45.4%) were responsive to the task according to the criterion described above. For each of those contacts, we calculated the time it took for the signal to reach 75% of the maximum ERP (for that contact). To account for different processing speeds among patients (i.e., measured by response time), we normalized the time to the leading edge of peak activation to each patient’s mean response time for each contact. This followed the same process as calculation of DES-Picture Onset-Asynchrony. To focus the analysis on neurophysiological activity corresponding to lexical access and word production processing (and not post–articulatory somatosensory and auditory feedback), only contacts with a time-to-peak signal that occurred before the average response time (for that subject) were included in analyses (665/690; 96.3%). We repeated this process for gamma-band signal analysis.

Visualization in the MNI 152 template space was accomplished by first projecting the time to the leading edge of peak activation for each contact, in all voxels within a 10-mm radius, weighted by the inverse of the Euclidean distance (1/*d*) of each voxel to the contact. Values from multiple nearby contacts were averaged at each voxel, resulting in a smooth functional map of time-to-peak SEEG signal (Fig. 3D). A 3D rendering of the MNI 152 template (left hemisphere) was used to visualize the results, using Matlab scripts in conjunction with BrainVoyager and BVQX tools. Maximum values up to 6 mm from the cortical surface were displayed on the cortical surface, followed by linear interpolation and kernel smoothing for visualization.
