# Supplementary material for: Causal parametric language mapping with electrical stimulation during awake neurosurgery
Source: Sci Adv. 2026 Feb 25;12(9):eadw1599. doi: 10.1126/sciadv.adw1599 (PMC12935032; doi:10.1126/sciadv.adw1599)
Supplement: Supplementary file 1 — Supplementary Text Figs. S1 to S5 Tables S1 to S4 Legends for movies S1 and S2 [file sciadv.adw1599_sm.pdf]

Science Advances
AAAS

Supplementary Materials for

Causal parametric language mapping with electrical stimulation during awake neurosurgery

J. Raouf Belkhir *et al.*

Corresponding author: Bradford Z. Mahon, bmahon@andrew.cmu.edu

Sci. Adv. **12**, eadw1599 (2026)
DOI: 10.1126/sciadv.adw1599

The PDF file includes:

Supplementary Text
Figs. S1 to S5
Tables S1 to S4
Legends for movies S1 and S2

Other Supplementary Material for this manuscript includes the following:

Movies S1 and S2

Supplementary Text

Predicting response times on negative mapping trials (Fig. 5)

We employed Leave-One-Out Cross-Validation (LOOCV) within a linear multiple regression framework to test whether DES parameters can predict response times on correct trials. Below, we outline the models assessed in 2 separate approaches.

1. **Model 1**: This model predicted response time based on DES Duration.
2. **Model 2**: This model predicted response time for left-out trials based on the absolute value of the difference between DES-Picture Onset-Asynchrony for the to-be-predicted trial, and the DES-Picture Onset-Asynchrony that resulted in the greatest delay in response time for the training data.
3. **Model 3**: This model was the same as Model 2, with the addition of DES duration.
4. **Model 4**: This model was the same as Model 3, with the addition of DES amperage.

In order to assess the predictive power of DES independent of spatial factors, we first assessed each model across the entire dataset. For each model, we used the training data (which is agnostic to DES location) to predict response time on the left-out trial and iterated this process over all trials.

In order to assess the predictive power of DES while incorporating spatial information, we assessed each model within a searchlight approach. For each searchlight, we applied the same models described above (Models 1-4) to each left-out trial that intersected with the searchlight. After iterating through all searchlights, we averaged the predicted response times for each trial, producing a single predicted response time for each model. To ensure adequate training data, we required a minimum of 10 trials for model fitting (i.e., at least 11 trials within a searchlight). Due to this trial threshold, not all DES events were included, leading to a reduced sample size relative to the whole-dataset LOOCV (604 trials vs. 679 trials).

Linear regression was then used to compare predicted and actual response times. In order to ensure that results of regression analysis were not driven by outliers, predicted response times more than 3 standard deviations from the mean (for each model), were removed from analysis. Since DES amplitude was not available for every DES event, this further reduced the sample size for model 4. Across all 8 models, generated response times significantly predicted observed response times (table S4; all $p<0.005$).

In order to assess whether models generated in a location specific manner fit the real data *significantly* better than models that are agnostic to DES location, we extracted the root squared mean error (RSME; as our measure of model fit on training data) for each regression, in each fold of the cross validation across both approaches. Across all 4 models, models performed significantly better when computed within the searchlight approach compared to when they are agnostic to DES location (table S4; $V>=145600$, $p<0.0001$; Wilcoxon matched-pairs ranked test).

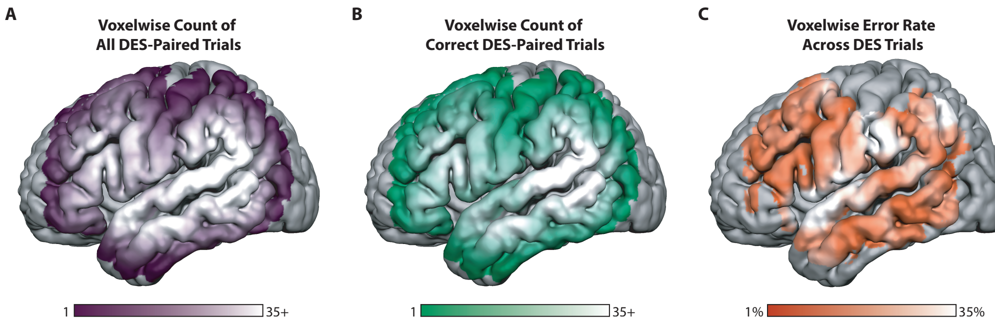

Fig. S1. Voxelwise mapping of DES trial distribution and error rates using a searchlight analysis.
To assess regional effects of direct electrical stimulation (DES) on behavior, we developed and implemented a searchlight analysis that aggregated local trial outcomes across a 5×5×5 voxel cube (15 mm^3) centered at each voxel of the MNI template brain. Analyses within this framework were constrained to sites with at least 10 DES events across participants. **(A)** Voxelwise count of all DES trials across analyses. **(B)** Voxelwise count of correct DES trials used for response time analysis. (C) Voxelwise error rate, calculated as the percentage of DES trials within each searchlight that resulted in an overt error. Maps are displayed on the MNI ICBM152 cortical surface. See Fig. 2E for statistical analysis of elevated error rates ($\chi^2 > 3.8$, $p < 0.05$).

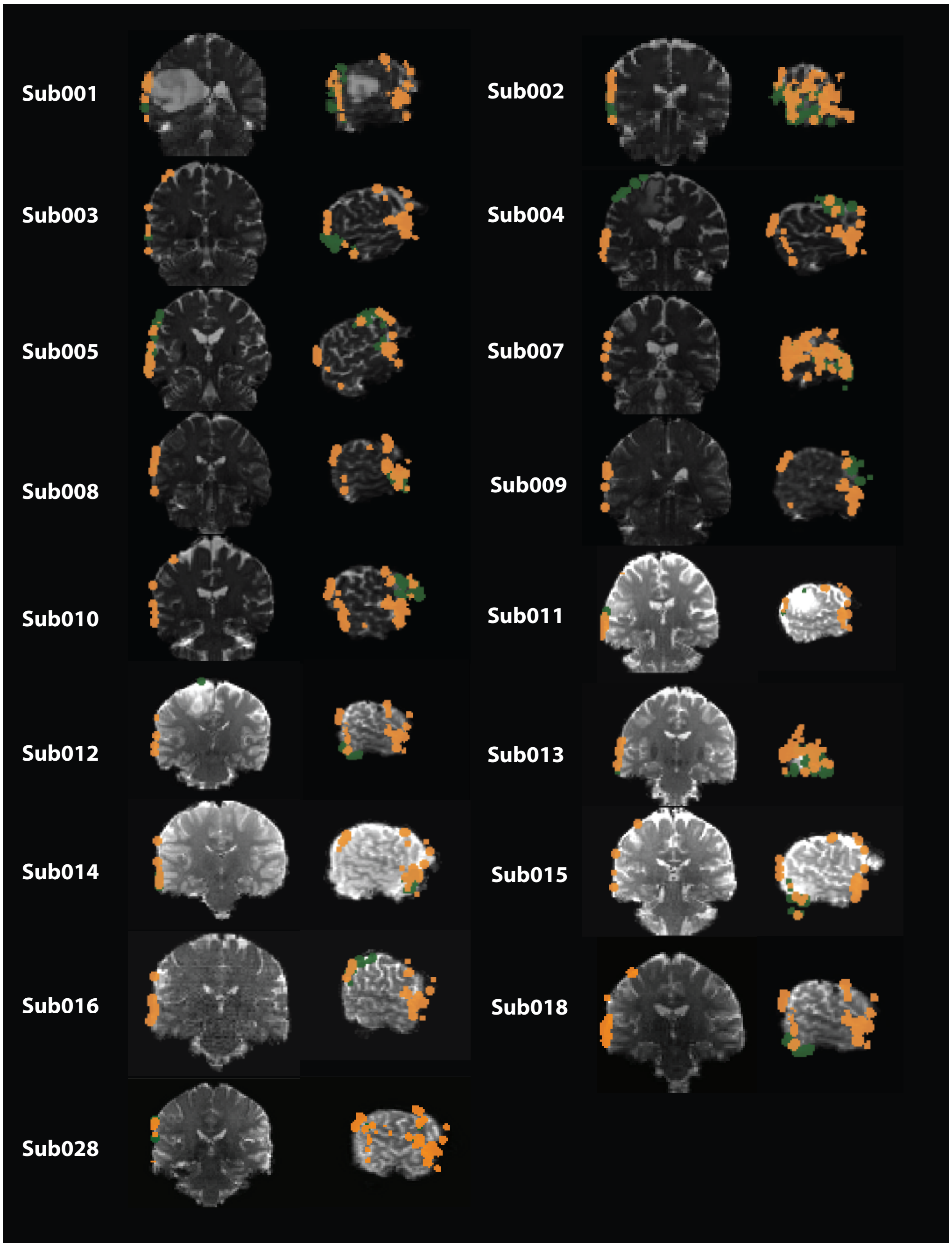

Fig. S2. Subject-specific tractography seeds and targets used for connectivity-to-error analysis. For each participant, probabilistic tractography was performed in subject-native diffusion space between stimulation sites from correct trials (5mm radius spherical seed masks; green) and the nearest error site(s), either within the same subject or across other subjects (orange masks). A separate within-subject analysis was conducted using an anatomically defined vSMC mask as the tractography target. Masks were transformed to diffusion space using FSL tools (see Methods), and streamlines were propagated with 5,000 samples per voxel for the connectivity-to-error analysis and 10,000 for the vSMC analysis. All masks are shown overlaid on each subject's b=0 diffusion reference image.

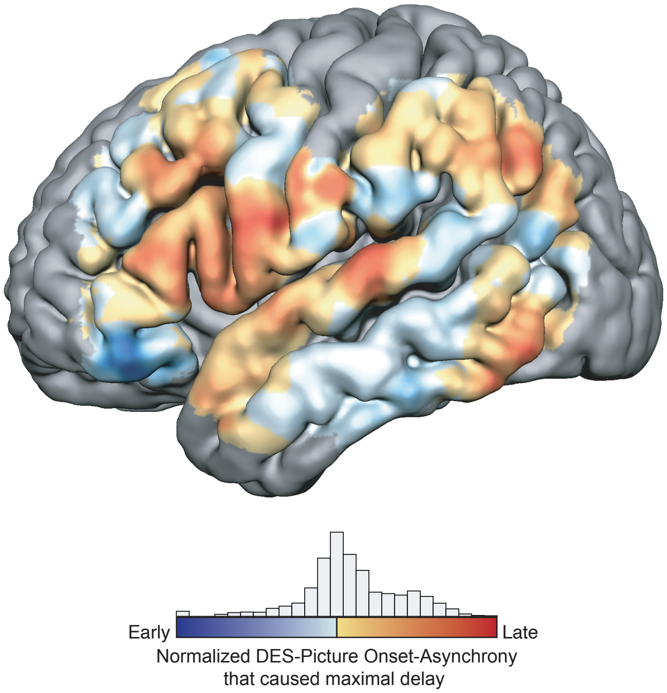

Fig. S3. Control analysis for interaction between location and maximum effect of DES-Picture Onset-Asynchrony. For each searchlight, the median DES-Picture Onset Asynchrony was calculated across all accurate trials within that searchlight. This median value was then subtracted from the DES-Picture Onset Asynchrony associated with the largest disruption in response time for the same searchlight. This normalization step accounts for potential clinician-related variability in timing across different regions. The interaction between location and the timing of the maximum DES effect on response time remained robust following normalization.

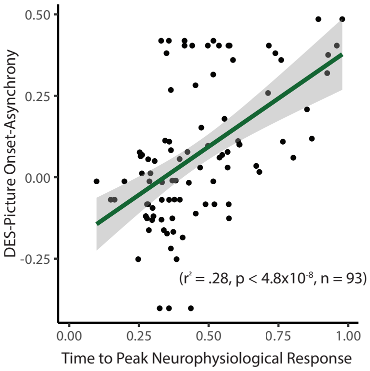

Fig. S4. Peak neurophysiological response timing predicts the behavioral impact of DES. A linear regression analysis found that the time to peak neurophysiological response (collected from intracranial recordings in a cohort of epilepsy patients) significantly predicted the value of DES-Picture Onset-Asynchrony (collected from a separate cohort undergoing awake language mapping) that was associated with the greatest slowing in response times during picture-naming ($R^2 = 0.281$; $F(1,91) = 35.6$, $p < 0.0001$). Each point reflects a single searchlight-defined location in the brain, with neurophysiological timing based on event-related potentials (ERPs). Regions where peak ERP responses occurred later also required later delivery of DES to maximally disrupt behavior. This cross-modal, cross-cohort alignment supports the interpretation that DES-Picture Onset-Asynchrony tracks architecturally successive stages of processing during language production. Results were robust to alternative measures and tests of covariance, including a rank-based correlation ($\rho=0.52, p<0.0001$) and a parallel analysis using gamma-band activity ($R^2 = 0.164$, $F(1,53)=10.4$, $p<0.003$).

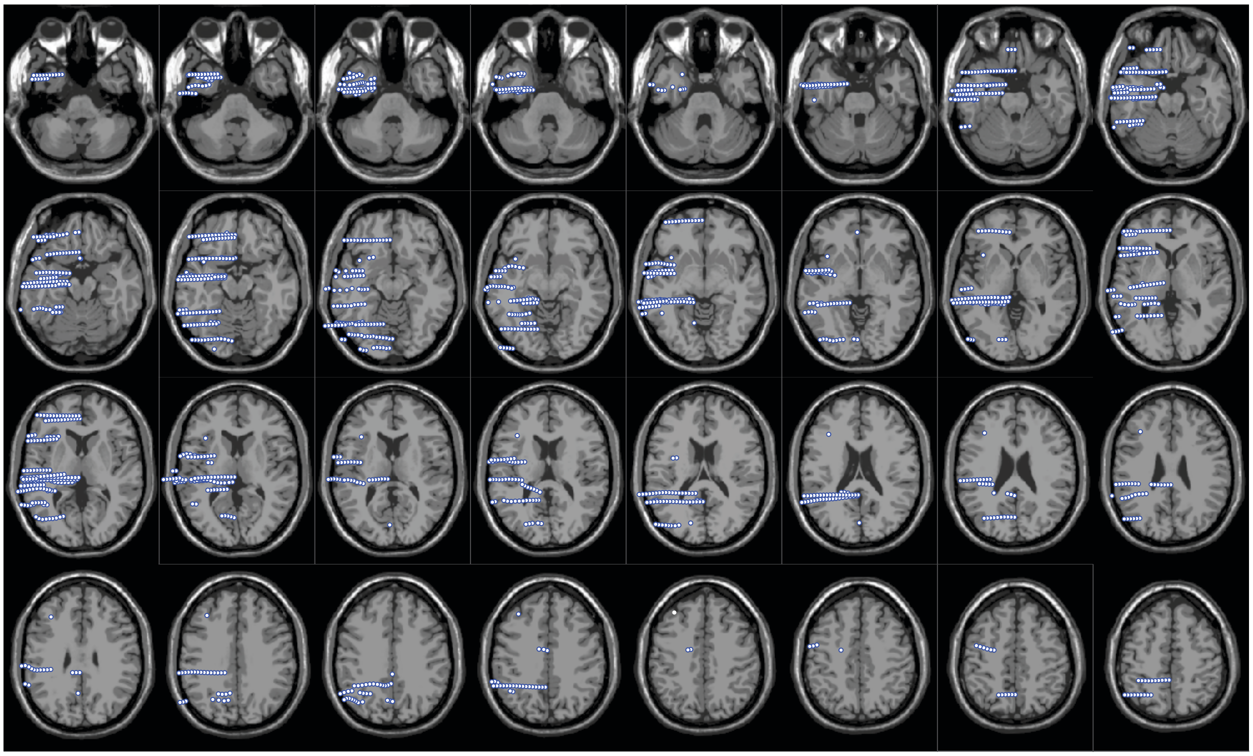

Fig. S5. Distribution of stereoencephalographic (SEEG) contacts in the left hemisphere visualized in MNI template space. Anatomical locations (coordinates) of stereoencephalographic (SEEG) electrode contacts in the left hemisphere across all analyzed patient participants (n=11) were reconstructed using Brainstorm and transformed to MNI template space (MNI ICBM 152) for group level analyses.

Table S1. Demographic, diagnostic, and lesion characteristics of the awake neurosurgery patient cohort

Subject ID	Age	Sex	Diagnosis	Pathology	Lesion Focus (size)
Sub001	34	M	LG Glioma	Astrocytoma IDH wt (+)	Inferior Parietal (74908 mm^3)
Sub002	58	F	HG Glioma	Glioblastoma IDH wt (+)	Posterior Temporal (11801 mm^3)
Sub003	44	M	Venous Malformation	-	Posterior Temporal (10130 mm^3)
Sub004	72	M	HG Glioma	Astrocytoma IDH wt (+)	Superior Frontal (79084 mm^3)
Sub005	75	M	HG Glioma	Astrocytoma IDH wt (+)	Anterior Temporal (34734 mm^3)
Sub006	79	F	LG Glioma	Astrocytoma IDH wt (+)	Medial Temporal (19704 mm^3)
Sub007	45	F	Cavernous Malformation	-	Anterior Temporal (183 mm^3)
Sub008	32	M	LG Glioma	Astrocytoma IDH wt (+)	Medial Temporal (8505 mm^3)
Sub009	42	M	HG Glioma	Astrocytoma IDH wt (-)	Inferior Frontal (71566 mm^3)
Sub010	70	F	LG Glioma	Oligodendroglioma IDH wt (-)	Middle Frontal (25170 mm^3)
Sub011	74	M	HG Glioma	Oligodendroglioma IDH wt (-)	Inferior Parietal (42216 mm^3)
Sub012	52	M	LG Glioma	Oligodendroglioma IDH wt (-)	Posterior Fronal (67034 mm^3)
Sub013	47	F	Venous Malformation	-	Posterior Temporal (7833 mm^3)
Sub014	35	M	LG Glioma	Ganglioglioma IDH wt (+)	Inferior Temporal (20878 mm^3)
Sub015	61	M	LG Glioma	Oligodendroglioma IDH wt (-)	Inferior Temporal (9308 mm^3)
Sub016	35	M	LG Glioma	Oligodendroglioma IDH wt (-)	Superior Parietal (19057 mm^3)
Sub017	68	M	HG Glioma	Glioblastoma IDH wt (-)	Middle Temporal (20064 mm^3)
Sub018	24	M	Refractory Epilepsy	-	Inferior Temporal
Sub028	27	M	Refractory Epilepsy	-	Anterior Temporal

Table S2. Task and stimulation parameters for the awake surgery patient cohort

Subject ID	ITI (ms)	Recorded Trials (% invalid)	Baseline Trials (% error)	DES Trials (% error)	Amperage (mA)	DES Duration (ms)	DES timing (ms)
Sub001	4300	127 (6.3)	71 (4.2)	48 (4.2)	2-4	2967	287
Sub002	4300	206 (3.4)	109 (2.8)	90 (10)	2.5-3.5	2675	-84
Sub003	4300	172 (3.5)	105 (7.6)	61 (11.5)	2.5-3	2726	-55
Sub004	6300	141 (20.6)	18 (16.7)	94 (11.5)	1	2529	234
Sub005	4300	195 (15.4)	99 (5.1)	66 (6.1)	-	3057	712
Sub006	4300	139 (16.5)	69 (8.7)	47 (17)	2-3	2906	56
Sub007	4300	181 (4.4)	120 (3.3)	53 (28.3)	6-8	2594	-143
Sub008	4300	176 (33.5)	85 (14.1)	32 (46.9)	5-9	2837	257
Sub009	4300	107 (0.9)	62 (1.6)	44 (0)	2.5	2082	5
Sub010	6300	51 (9.8)	21 (4.8)	25 (12)	-	2751	-304
Sub011	6300	45 (4.4)	17 (17.6)	26 (34.6)	-	3371	142
Sub012	5300	35 (2.9)	15 (0)	19 (0)	-	2774	-6
Sub013	6300	47 (12.8)	14 (0)	27 (0)	-	3566	-41
Sub014	6300	138 (5.8)	76 (11.8)	54 (20.4)	2.5	3671	120
Sub015	6000	140 (10.7)	61 (16.4)	64 (18.8)	5-8	3671	-410
Sub016	5000	56 (5.4)	16 (0)	37 (0)	4-7	2965	-408
Sub017	7000	109 (26.6)	46 (26.1)	34 (26.5)	6	1903	-651
Sub018	3500	274 (4.4)	231 (8.2)	31 (16.1)	3-4	3910	-425
Sub028	4000	159 (6.3)	134 (5.2)	15 (20)	-	2469	-6

Table S3. Demographic and clinical epilepsy characteristics of the stereoencephalographic patient cohort

Subject ID	Age	Gender	Period of Epilepsy Onset	Epilepsy Duration	Age of Onset	ILAE Focal Seizure Onset	ILAE Focal Seizure Awareness	Baseline Seizure Freq.
Sub019	34	F	Adolescence (11-19yrs)	14 years	17	Nonmotor onset	Impaired awareness	>=1/month
Sub020	40	M	Adulthood (>20yrs)	4 years	34	Motor onset	Impaired awareness	>=1/month
Sub021	31	M	Adulthood (>20yrs)	4 years	26	Motor onset	Aware	>=1/week
Sub022	70	M	Adulthood (>20yrs)	27 years	41	Motor onset	Impaired awareness	>=1/3months
Sub023	44	F	Adulthood (>20yrs)	9 years	32	Nonmotor onset	Impaired awareness	>=1/3months
Sub024	58	F	Adulthood (>20yrs)	35 years	22	Nonmotor onset	Impaired awareness	>=1/week
Sub025	55	M	Adulthood (>20yrs)	30 years	24	Nonmotor onset	Impaired awareness	>=1/week
Sub026	63	F	Adulthood (>20yrs)	2 years	60	Nonmotor onset	Impaired awareness	>=1/week
Sub027	24	M	Adolescence (11-19yrs)	5 years	18	Nonmotor onset	Impaired awareness	>=1/3months
Sub028	27	M	Adulthood (>20yrs)	7 years	20	Nonmotor onset	Impaired awareness	>=1/day
Sub029	58	F	Adulthood (>20yrs)	11 years	46	Nonmotor onset	Impaired awareness	>=1/week

Table S4. Extended analyses of location-agnostic and location-specific models predicting response times on negative mapping trials

	Across entire dataset				Location Specific					
**Model**	**n**	**R^2**	**p-value**	**mean RSME**	**n**	**R^2**	**p-value**	**mean RSME**	**V**	**p-value**
Model 1 (DES Duration)	674	0.321	<0.0001	452.5	601	0.348	<0.0001	403.6	154126	<0.0001
Model 2 (DES-Picture Onset Asynchrony)	671	0.012	<0.005	526.8	597	0.073	<0.0001	478.6	146500	<0.0001
Model 3 (DES Duration x DES-Picture Onset Asynchrony)	677	0.345	<0.0001	433.6	600	0.438	<0.0001	369.2	172807	<0.0001
Model 4 (DES Duration x DES-Picture Onset Asynchrony x DES Amperage)	444	0.412	<0.0001	402.6	391	0.542	<0.0001	299.6	157476	<0.0001

Movie S1. Selected examples of 'negative mapping' trials during awake language mapping with direct electrical stimulation, illustrating how patient behavior is parameterized for Causal Parametric Mapping. This video shows multiple picture-naming trials from patients undergoing awake neurosurgery, during which direct electrical stimulation (DES) is delivered as participants name visually presented objects. The upper-left panel displays the surgical field, stimulation site, and target picture for each trial, with the patient's naming response (audio and mouth movements; faces blurred for privacy) shown concurrently. The upper-right panels show response times for each trial plotted against DES picture-onset asynchrony and DES duration, with separate lines of best fit for each participant (LOWESS for DES picture-onset asynchrony; linear regression for DES duration). The lower panels illustrate the neurocognitive stages of picture naming, the conceptual framework showing how DES parametrically modulates performance across processing stages, the stimulation site rendered on an MNI template cortical surface, and a time-aligned bar depicting the onset, duration, and response time associated with DES relative to picture onset. The video can be viewed at: https://youtu.be/42bOZzlDfTk

Movie S2. Spatiotemporal evolution of task-related local field potentials during picture naming. The opening frame displays the full distribution of stereoencephalographic (SEEG) contacts from 11 participants rendered on an MNI template cortical surface. White spheres denote contacts with significant event-related potential (ERP) amplitude changes relative to baseline during picture naming, and black spheres denote contacts without significant change. The video then depicts the trial-averaged, time-resolved ERP waveforms (derived from baseline-corrected local field potentials) for significant contacts only. Sphere color intensity reflects each contact's percent change from baseline (450-150 ms before picture onset), scaled to its own peak response. Sequences are first shown in real time aligned to picture onset (0 ms), followed by a slow-motion replay (30% real-time speed) to highlight the temporal progression of task-related cortical activity. The video can be viewed at: https://youtu.be/5KZe-7jIe0g
